# Biomimetic Antibacterial Gelatin Hydrogels with Multifunctional Properties for Biomedical Applications

**DOI:** 10.1021/acsami.3c10477

**Published:** 2023-11-17

**Authors:** Hengzhi Ruan, Marko Bek, Santosh Pandit, Alexandra Aulova, Jian Zhang, Philip Bjellheim, Martin Lovmar, Ivan Mijakovic, Roland Kádár

**Affiliations:** † Department of Industrial and Materials Science, Chalmers University of Technology, 412 96 Göteborg, Sweden; ‡ Department of Biology and Biological Engineering, Chalmers University of Technology, 412 96 Göteborg, Sweden; § Welspect AB, 431 21 Mölndal, Sweden; ∥ The Novo Nordisk Foundation Center for Biosustainability, Technical University of Denmark, 2800 Kongens Lyngby, Denmark

**Keywords:** gelatin, antibacterial materials, hydrogel coating, multifunctional performance, biomimetic strategy

## Abstract

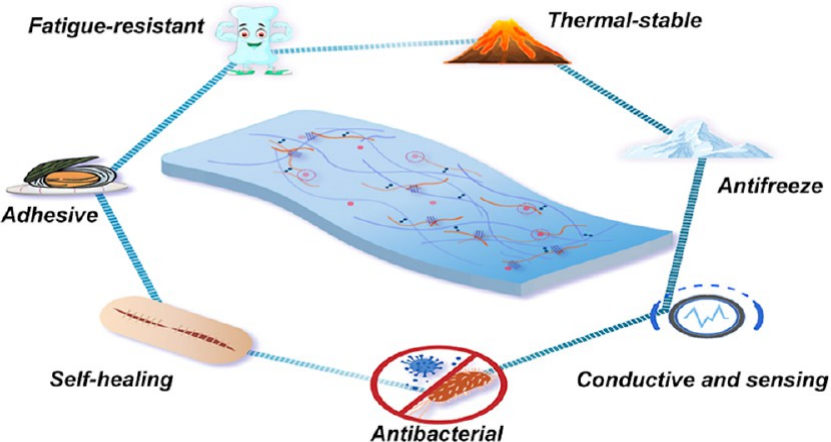

A facile novel approach of introducing dopamine and [2-(methacryloyloxy) ethyl] dimethyl-(3-sulfopropyl) ammonium hydroxide via dopamine-triggered in situ synthesis into gelatin hydrogels in the presence of ZnSO_4_ is presented in this study. Remarkably, the resulting hydrogels showed 99.99 and 100% antibacterial efficiency against Gram-positive and Gram-negative bacteria, respectively, making them the highest performing surfaces in their class. Furthermore, the hydrogels showed adhesive properties, self-healing ability, antifreeze properties, electrical conductivity, fatigue resistance, and mechanical stability from −100 to 80 °C. The added multifunctional performance overcomes several disadvantages of gelatin-based hydrogels such as poor mechanical properties and limited thermostability. Overall, the newly developed hydrogels show significant potential for numerous biomedical applications, such as wearable monitoring sensors and antibacterial coatings.

## 1 Introduction

Hydrogels are a unique class of materials with hydrophilic, self-supporting, three-dimensional networks.^1^ The presence of physical or chemical cross-links in the network leads to their structural integrity and results in different properties. Meanwhile, the structure of hydrogels can be similar to the natural extracellular matrix, which allows the diffusion and the attachment of molecules and cells.^2^ Therefore, hydrogels have received extraordinary attention in biomedical applications, such as drug delivery, wound dressing, tissue engineering, personalized sensors, and medical device coatings.^2,3^

One of the common materials to fabricate hydrogels for biomedical applications is gelatin, a degradation product of collagen with many excellent biological activities such as biodegradability, biocompatibility, and nonimmunogenicity.^4^ Furthermore, the rheological characteristics of gelatin can be used to adjust the solution viscosity, and gelatin itself can form physically cross-linked hydrogel below the sol–gel transition temperature.^3^ With these advantages, gelatin-based hydrogels are widely used in the manufacturing of contact lenses, scaffolds for tissue engineering, and drug delivery matrices.^5^ The low cost and commercial availability of gelatin provide opportunities to achieve large-scale production of, e.g., coatings. However, it is still challenging to fabricate gelatin-based hydrogels that can simultaneously meet the demands of practical applications, such as tuneable mechanical performance, electrical conductivity, adhesion, stability in a wide range of temperatures, cytocompatibility, self-healing, antimicrobial properties, etc. Additionally, gelatin properties such as gel strength and thermostability depend highly on the amino acid composition and molecular weight distribution, which can vary largely based on processing conditions.^6^ Thus, both enhancing the multifunctional properties and extending their functionality are current topics of interest. Gelatin-based hydrogels generally suffer from lack of stability in mechanical properties at and above physiological temperatures.^3^ Furthermore, due to their large water content, hydrogels tend to lose their ionic transport and mechanical performance if subjected to freezing temperatures. Pure gelatin is easily spoiled by bacteria and unable to prevent biofilm formation.^7^ Overall, there are always trade-offs between multifunctionality and performance. For example, Luo et al. fabricated an injectable gelatin hydrogel for hemorrhage control with good cytocompatibility while other desirable properties such as adhesion and self-healing were not found in the materials;^8^ Gelatin-based hydrogel electrodes with conformal skin adhesion and uniform electrical conductivity could be used for electrocardiography monitoring, whereas the mechanical properties are not tough enough considering long-term use.^9^

Nature is an important source of inspiration for the development of new technologies and advanced materials. A typical example is the construction of remarkable surface adhesion to various materials through mussel chemistry.^10^ It has been found by Messersmith et al. that mussels exhibit strong adhesion in seawater by the mussel foot proteins containing 3,4-dihydroxyphenylalanine (DOPA).^11^ The catechol group on the side chains of DOPA is regarded as the main reason for the strong reaction with various substrate surfaces via noncovalent interactions and chemical cross-linking.^12^ Meanwhile, there are also other residues in mussel foot proteins including hydrophobic and charged moieties that improve wet adhesion through the control of hydrophobic, cation−π, or electrostatic interactions.^13^ These properties have made DOPA and its derivatives useful as essential building blocks for engineering new hydrogels.^14^ Zwitterionic monomers are another type of hydrogel building blocks, with both positive and negative charges, that can firmly bind with water molecules through the electrostatic interaction, which increases the saturated water content and improves hydrogels with electrical conductivity.^15^ Many plants such as *Arabidopsis* rely on zwitterionic osmolytes including betaine and proline, to avoid low-temperature frostbite^16^ because these zwitterionic small molecules can be used as hydrogen bond donors to combine with water molecules, inhibiting the formation of ice crystals at subzero temperatures.^17^ Among them, [2-(methacryloyloxy) ethyl] dimethyl-(3-sulfopropyl) ammonium hydroxide (SBMA) has been proven to repel the adhesion of bacteria and proteins by generating a dense hydration layer,^18,19^ which largely expands the applications of hydrogel in biomedicine.

In addition, considerable amount of research has focused on dopamine-initiated synthesis.^20^ One relevant aspect is that dopamine-triggered reactions with zwitterionic monomers can greatly simplify the synthesis steps and enrich the functionality of hydrogel materials.^21^ However, in this way strong hydrogen and ionic bonds between long chains tend to result in low network flexibility, and therefore, it is difficult to break the alignment of small water molecules at low temperatures, which limits their use.^22^ Ion specificity effects (Hofmeister series), via, e.g., carbonate and sulfate ions, affect the mechanical properties of gelatin-based hydrogels, and these inorganic particles can act as reversible cross-linkers in gelatin networks. Meanwhile, typical strategies to endow hydrogels with antibacterial properties rely on antimicrobial metal ions such as silver, zinc, and copper.^23^ Thus, when combined with zwitterionic SBMA, a novel hydrogel that can not only kill bacteria but also repel bacterial attachment could create synergetic antibacterial effects, an important aspect to tackle healthcare-associated infections.

In this study, we present a simple approach to synthesize versatile gelatin-based hybrid hydrogels with antibacterial activities, adhesive properties, self-healing ability, electrical conductivity, fatigue-resistance, and mechanical stability from −100 to 80 °C. The composition of the hydrogels is unique and was tailored to achieve a broad spectrum of multifunctional properties. Dopamine was chosen as the monomer because it provides excellent adhesion, allowing hydrogel materials to adhere to different surfaces. Furthermore, it can act as a reaction initiator by generating free radicals through self-oxidation. SBMA was chosen as a comonomer due to its high hydrophilicity and antifouling properties, while the formation of ionic bonds should improve the electrical conductivity of the hydrogels. In addition, the oligomer of SBMA and dopamine can prevent the hydrogels from freezing through hydrogen bonds and electrostatic interactions and further enhance the stability of the hydrogel at high temperature via multiple supramolecular forces. By the addition of zinc sulfate, the enhancing of adhesion, conductivity, and antibacterial properties of the hydrogels was targeted. Combined with the hydrogen bonding brought by gelatin, we hypothesized that the hydrogels can be endowed with self-healing ability. Overall, the combined significant enhancements in multifunctional properties show that the novel hydrogels could have a high potential for a number of biomedical applications, such as a wearable biosensor to detect the motion of the human body and as antibacterial coatings.

## 2 Experimental Section

### 2.1 Materials

Gelatin (gel strength 300, Type A), SBMA, dopamine hydrochloride, zinc sulfate heptahydrate, lysogeny broth (LB broth), and tryptic soy broth (TSB broth) were purchased from Sigma-Aldrich (Burlington, MA, USA).

### 2.2 Preparation of the Hydrogels

For gelatin hydrogel samples, gelatin and deionized water were ultrasonically mixed at 60 °C for 20 min, and the solution was poured into a mold followed by cross-linking in the fridge at 3 °C for 3 h. For GZ5 and GZ10 samples, gelatin was added into zinc sulfate solution (0, 5, 10, and 20%) and then mixed ultrasonically at 60 °C for 20 min, followed by cross-linking in the fridge for 3 h. By adjusting the concentration of zinc sulfate solution, different GSD/GSDZ hydrogels were synthesized. Gelatin, SBMA, dopamine hydrochloride, and deionized water or zinc sulfate solution were mixed and stirred for 30 min at 50 °C. The obtained mixture in solution was ultrasonically homogenized and poured into a mold in a closed box to ensure the cross-linking environment constant, before being heated at 60 °C for 12 h to trigger the polymerization of SBMA and dopamine. The hydrogels with different mass concentrations of zinc sulfate solution (0, 5, 10, and 20%) were marked as GSD, GSDZ5, GSDZ10, and GSDZ20. The hydrogels with lower SBMA concentrations were marked as GS0DZ5, GS1DZ5, and GS2DZ5. The detailed feed ratio is shown in Table 1.

**Table 1:** Samples and Their Composition

sample name	gelatin (g)	dopamine (g)	SBMA (g)	5% ZnSO_4_ solution (mL)	10% ZnSO_4_ solution (mL)	20% ZnSO_4_ solution (mL)	water (mL)
gelatin gel	1.5	0	0				8.5
GZ5	1.5	0	0	8.5			
GZ10	1.5	0	0		8.5		
GSD	1.5	1	1.5				6
GSDZ5	1.5	1	1.5	6			
GSDZ10	1.5	1	1.5		6		
GSDZ20	1.5	1	1.5			6	
GS0DZ5	1.5	1	0	6			1.5
GS1DZ5	1.5	1	0.5	6			1
GS2DZ5	1.5	1	1	6			0.5

### 2.3 Fourier Transform Infrared Spectroscopy

Infrared spectra analysis of gelatin hydrogels as well as GSDZ5, GSDZ10, and GSDZ20 hydrogels for chemical characterization were conducted on an Alpha spectrometer (Bruker Hyperion3000/Vertex70v; Billerica, MA, USA) in the range of 4000–500 cm^–1^ resolution after 32 scans. Before the measurement, a background spectrum was measured. All measurements were performed in triplicate.

### 2.4 Gel Permeation Chromatography

After 12 h at 60 °C incubation with DI water or 5% zinc sulfate solution, the molecular weight of the SBMA and dopamine was determined using gel permeation chromatography (GPC, Agilent Technologies 1260 Infinity II equipped with a multi detector system, which includes a refractive index-detector, CA, USA). Samples were diluted to 5 mg/mL before injecting. The system was calibrated using poly(ethylene glycol)/oxide EasiVial Narrow Standards from Agilent. Data evaluation was performed using an Agilent GPC Software.

### 2.5 Scanning Electron Microscope

GSDZ10 hydrogel bulks were rinsed gently using DI water and immersed in DI water for 12 h. Then, the samples were shock-frozen in liquid nitrogen, followed by lyophilization in a freeze-drier. The dried samples were coated by gold with the thickness of 4 nm before SEM (Zeiss Ultra 55 FEG; Oberkochen, Germany) observation on the cross section of the specimens. GSDZ10 was chosen considering the moderate concentration of ZnSO_4_. For comparison, gelatin hydrogel bulks were treated and analyzed the same way.

### 2.6 Tensile Tests

All tensile tests were performed by using a Zwick/Roell Z1.0 (Ulm, Germany) universal testing machine equipped with a 100 N loading cell. For the tensile test, dumbbell-shaped samples (ISO 527-2-Type 5A) were tested with an initial distance of 30 mm between the two grips and a constant strain rate of 10 mm/min. All mechanical tests were performed at room temperature.

### 2.7 Fatigue Tests

To further investigate the fatigue behavior of materials, loading–unloading tests were performed using the same universal testing machine as for tensile tests. For the load–unload tests, GSDZ5 samples (ISO 527-2-Type 5A) were strained for ε = 13% with loading and unloading speed 10 mm/min for 10 times. The dissipated energy was assessed by integrating the area between the loading–unloading curves.

### 2.8 Adhesive Tests

Adhesive tests were performed using a rotational rheometer (Anton Paar MCR702; Graz, Austria) with a stainless-steel plate–plate geometry (25 mm diameter). A recently developed testing method, RheoTack, was for the first time used to investigate the adhesive properties of hydrogels since it could adjust retraction speed, compression force, and geometries to observe the adhesive phenomenon of samples.^24^ Combined with optical visualizations, the method could provide insights into the adhesion and detaching behavior of samples, for example, fibril formation. Prior to each test, the GSDZ hydrogels were dipped in water for a few seconds to ensure that the surface of the hydrogel is wet. For RheoTack,^24^ hydrogel samples with a diameter of 25 mm were placed on the bottom plate. The upper plate was moved downward with a speed of 0.1 mm/s until reaching a compression force of 0.5 N followed by a dwell time of 180 s to provide initial adhesion. Due to the viscoelastic (time-dependent) nature of the hydrogels, the adhesive force and energies are affected by the retraction speed, relative to the intrinsic relaxation of the material.^24^ Low retraction speeds allow more material relaxation under load, while high retraction speeds usually lead to more brittle cohesiveness or adhesive failure. Therefore, two different velocities for lifting the upper plate were selected (0.01 and 0.1 mm/s) to represent the adhesive properties of the hydrogels. The force and displacement of the material were recorded, and the adhesion energy was calculated. Adhesion energy is defined as the integral of the force–displacement curve until there is complete loss of adhesion.

### 2.9 Self-Healing Tests

An optical microscope (Zeiss Axioscope 5/7/Vario; Oberkochen, Germany) was employed to observe the self-healing process of hydrogels. For the qualification of the self-healing efficiency, a rectangular hydrogel sample (10 mm × 50 mm × 2 mm) was cut in two parts, and then, the two-halves were placed back together immediately without any additional stress and left under room temperature for 12 h. After a certain time, the hydrogel samples were transferred into an oven at 60 °C for further recovery. The self-healing ability of the hydrogels was evaluated by performing tensile tests. For this, the contrast and original hydrogel samples (ISO 527-2-Type 5A) were first tested following the same testing procedure as that for tensile tests. Afterward the samples with same compositions were cut into two pieces. One-half was stained with methylene blue, while the other half was stained with rhodamine B. The two cross sections of hydrogel samples were put back into the mold and transferred into an oven for 6 h. After healing, the tensile properties of samples were tested again.

### 2.10 Electrical and Sensing Tests

A rheo-dielectric setup mounted on the MCR702 rheometer already mentioned was used to investigate the self-healing process and sensing properties of the hydrogels. For self-healing, dielectric measurements were carried out in the (electrical) frequency range of 10 to 10^6^ Hz, using an impedance analyzer (Zurich Instruments MFIA; Zürich, Switzerland). The intact reference samples and the healing samples (25 mm in diameter) were sandwiched between two titanium electrodes (plate–plate measuring geometry with 25 mm in diameter) which were mounted on the rheometer and connected to the dielectric spectrometer through shielded cables. For sensing properties, a time sweep with constant normal force (2, 4, 6, 8, and 10 N) was conducted on the test samples. No shear loading was applied to the sample. While dielectric spectroscopy data can be the topic of extensive analysis, in this work we focus mainly on the conductive properties of the hydrogels. The alternating current complex conductivity is
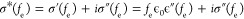
1where *f*_e_ is the electrical frequency, σ′ and σ″ are the real and imaginary parts of the complex conductivity, σ* is the complex conductivity, ϵ_0_ is the vacuum permittivity, and ϵ″ is the imaginary part of the dielectric function. The (DC) conductivity can be determined in the limit.
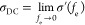
2

In addition to the rheo-dielectric setup, a wearable biosensor model was assembled by placing GSDZ20 hydrogel samples between two electrodes and fixed on the finger of a human hand model. Resistance in real-time was obtained from an electrochemical workstation with a constant voltage of 3 V and calculated by Ohm’s law.

### 2.11 Antifreeze and Thermal Stability Tests

For the dynamic mechanical properties of gelatin, GSD and GSDZ samples were measured using a rotational rheometer (Anton Paar, MCR702; Graz, Austria) in a separate motor-transducer configuration. The rheometer was equipped with a convection oven. Two sets of measuring geometries were used to adapt to the specific viscoelastic material response of the samples. For GSD and GSDZ samples, solid rectangular fixtures were adopted to avoid compliance error at low temperature, and the normal force was applied at −0.5 N to prevent sample buckling and to compensate for sample thermal expansion. For gelatin, plate–plate geometries (25 mm in diameter) were chosen with a normal force of 2 N to avoid sample slip at low temperatures. Dynamic temperature sweep tests were performed at constant strain amplitude (0.01%) and frequency (1 Hz) with the temperature ranging from 10 to −100 °C and a cooling rate of 5 °C/min. Rectangular test samples of approximately 2 mm thickness, 10 mm width, and 30 mm length were used. The effect of negative temperature on hydrogels was also studied based on differential scanning calorimetry (DSC) data.

To investigate the dynamic mechanical properties of the hydrogels, the same setup and parameters as used in antifreeze tests were adopted for thermal stability tests except for the temperature ramp from 10 to 80 °C with a heating rate of 5 °C/min on fresh test samples. Dynamic mechanical thermal analysis (DMTA) results were supported by DSC and thermal gravimetrical analysis (TGA) findings.

### 2.12 Thermal Characterization

Gelatin hydrogel, GSD, and GSDZ10 hydrogel samples were investigated by means of DSC2 (Mettler; Columbus, OH, USA) and TGA/DSC3+ (Mettler; Columbus, OH, USA) with heating/cooling rates of 10 °C/min. DSC tests were performed in the nitrogen atmosphere starting from room temperature and heating up to 60 °C, followed by cooling to −65 °C and second heating to 60 °C. TGA measurements were performed in air by heating from room temperature to 300 and 500 °C for gelatin hydrogel and GSDZ10 samples, respectively. Results of experiments were used for thermal stability and antifreeze analysis.

### 2.13 Rheological Tests

Rheological measurements on the precursor solution of GSDZ10 hydrogels, considering the moderate concentration of ZnSO_4_, were performed using the Anton Paar MCR702 rheometer with a plate–plate measuring geometry (25 mm in diameter). The time sweep experiments were made at strain of 0.5% at a constant frequency (0.05, 0.1, 0.5, 1, and 10 rad/s) for 20 min at 25 °C.

### 2.14 Preparation of the Coatings of GSD and GSDZ Hydrogels

The targeted substrates (glass slides, 75 × 25 × 1 mm and stainless steel, diameter 23 mm) were cleaned by washing with ethanol and water, followed by drying in an oven at 40 °C. Subsequently, the clean samples were immersed into homogeneous GSD/GSDZ solutions and withdrawn slowly to form a precoating layer on the surface of samples, followed by drying at 60 °C for 12 h.

### 2.15 Atomic Force Microscopy

The microscopic morphology of the coatings based on GSDZ5, GSDZ10, and GSDZ20 hydrogels was investigated by using AFM (NT-MDT NTEGRA; Russian Federation). Glass slides were chosen as the substrate to explore the microscopic morphology of GSDZ hydrogel coatings fabricated by different concentrations of zinc sulfate solution (5, 10, and 20%) to investigate the effects of zinc sulfate on coating roughness. The images were captured at a scan rate of 2.93 Hz and have dimensions of 5 μm × 5 μm. The data analysis was performed with Gwyddion Software.

### 2.16 Antibacterial Tests

Two different bacterial strains, *Escherichia coli* UTI89 (*E. coli*; Gram negative) and *Staphylococcus aureus* CCUG10778 (*S. aureus*; Gram positive), were used to examine the antimicrobial potential of produced hydrogels. *E. coli* was grown in LB broth, and *S. aureus* was grown using TSB broth. The effectiveness of GSDZ-coated samples to prevent biofilm formation by *E. coli* and *S. aureus* was quantified as described below. The respective overnight bacterial cultures were diluted to obtain a final inoculum of (2–5) × 10^6^ cfu/mL and cultured on the surface of GSDZ coatings, followed by incubation for 24 h at 37 °C to grow biofilms of respective bacterial strain. After the 24 h, biofilms were rinsed twice with sterile water and collected in 5 mL of 0.89% of sodium chloride. Biofilms were dispersed and homogenized by sonication (30 s). The homogenized biofilm suspensions (100 μL) were serially diluted and placed on agar plates and incubated at 37 °C for 24 h, and colonies were counted. Furthermore, SEM was used to visualize and confirm the antifouling results obtained from cfu counting. The samples for the SEM examination were prepared, as previously described.^25^ The biofilms grown on control samples and GSD and GSDZ5 hydrogel coatings were fixed with 3% glutaraldehyde for 2 h. The fixed biofilms samples were dehydrated by using a graded series of ethanol concentrations (40, 50, 60, 70, 80, and 90%) for 15 min each and with absolute ethanol for 20 min. All the dehydrated biofilms were dried overnight at room temperature and sputter coated with a thin layer of gold (ca. 5 nm setting) before SEM imaging (Zeiss Ultra 55 FEG; Germany).

## 3 Results and Discussion

### 3.1 Preparation of the Hydrogels and Chemical Characterization

**Figure 1:**
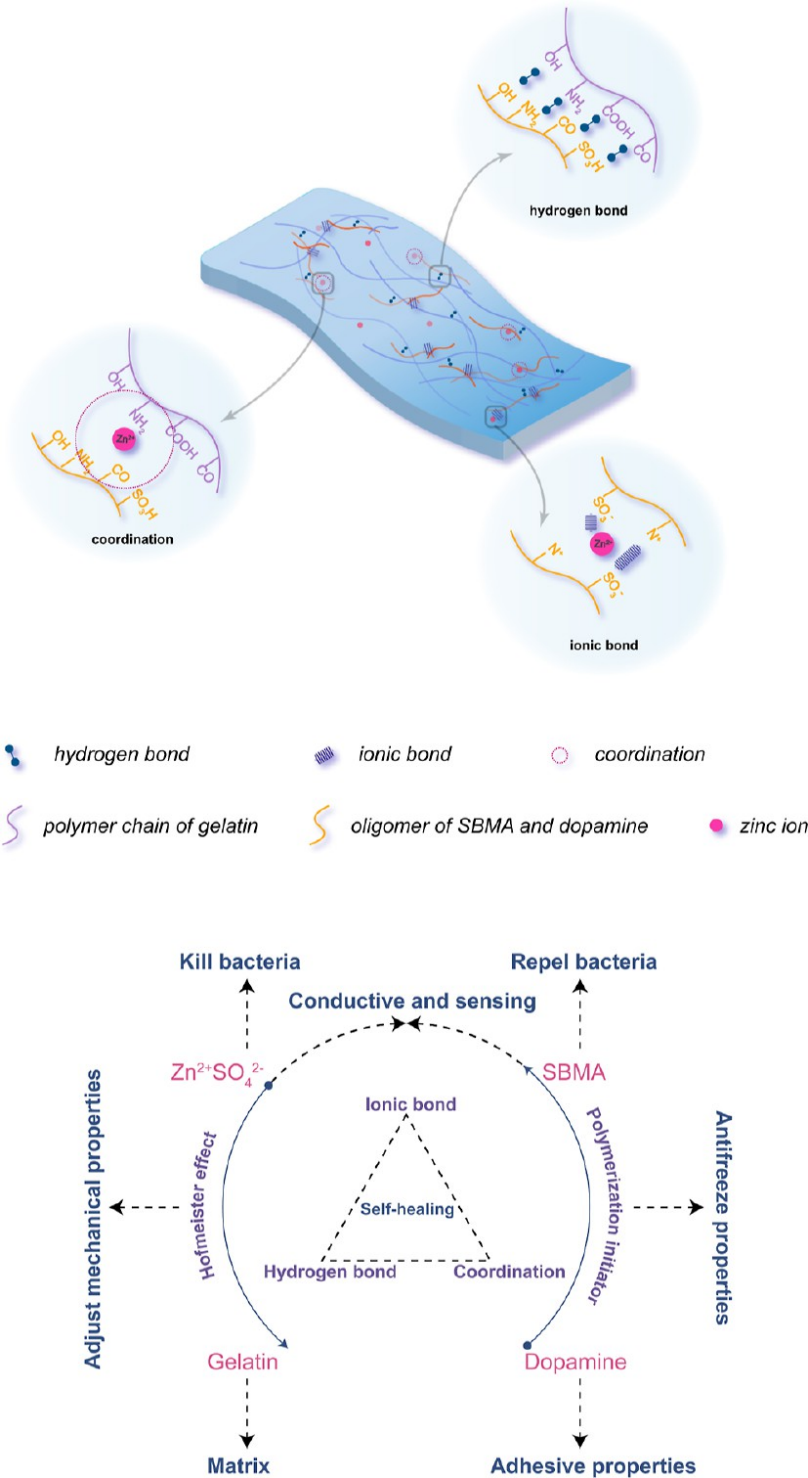
Schematic diagram of GSDZ hydrogels with multifunctional properties.

A multifunctional hydrogel material was prepared by introducing short oligomer chains of SBMA and dopamine together with zinc sulfate into hydrogels constructed from physically cross-linked gelatin long chains (Figure 1). Afterward, the gelatin was dissolved in preheated deionized water or zinc sulfate solution and SBMA. Dopamine monomers were added to the gelatin solution, where it acted as both the polymerization initiator and cross-linking meditator,^21^ followed by gelation at 60 °C to obtain GSD/GSDZ hydrogels. As illustrated in Figure 1, SBMA-dopamine oligomers can interact with the gelatin network through hydrogen bonds and can form ionic bonds with zinc ions and other oligomers. Meanwhile, the coordination effect can happen between zinc ions and dopamine.

As shown in the following sections, numerous supramolecular interactions ensure the self-healing properties of hydrogel materials and stability at a high temperature of 80 °C, which far exceeds the sol–gel transition temperature of gelatin. In addition, the adjustment of adhesive and mechanical properties was achieved via changing the content of zinc sulfate. It is worth noting that the oligomers can not only inhibit ice growth through hydrogen bonds or electrostatic interactions, endowing the hydrogel with antifreeze properties, but also provide the adhesion of the hydrogel materials through the catechol groups on the dopamine. The oligomers also act as a complement to the relatively rigid backbone of gelatin and impart the desired mechanical and rheological properties to the GSD/GSDZ hybrid hydrogels. Furthermore, by taking advantage of the greater mobility of oligomers compared to long polymer chains, the ionic bonds provided by oligomers could lead to electrical conductivity, which is further strengthened by introducing zinc sulfate.

### 3.2 FTIR and GPC Analysis of the Prepared Hydrogels

**Figure 2:**
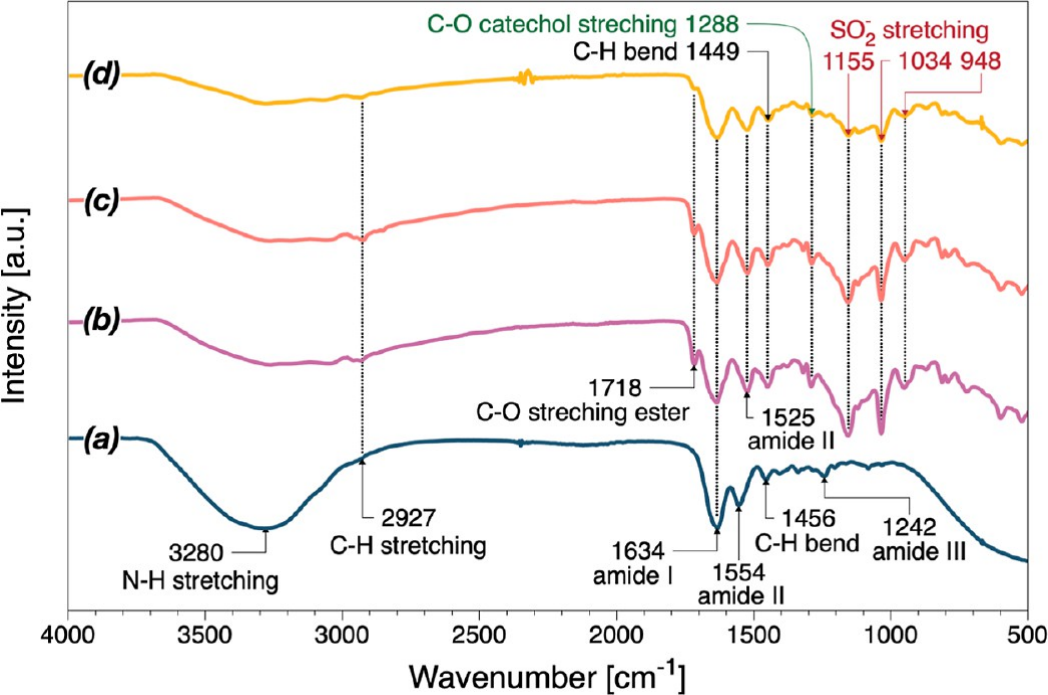
FTIR spectra of (a) gelatin, (b) GSDZ5, (c) GSDZ10, and (d) GSDZ20 hydrogel samples.

The functional groups of the hydrogel were analyzed using FTIR spectroscopy. As shown in Figure 2, for gelatin hydrogel samples, the absorption bands at 3280 and 2927 cm^–1^ were assigned to N–H stretching and C–H stretching vibration, respectively. Moreover, the peaks at 1634, 1554, and 1242 cm^–1^ were attributed to amide I, amide II, and amide III, respectively. The peak at 1456 cm^–1^ was assigned to C–H bending, which is in agreement with the previous reports.^26,27^ For GSDZ hydrogel samples (spectra a, b, c), the absorption peak at 1155, 1034, and 948 cm^–1^ were assigned to the stretching vibration bands of the S=O group in SBMA. The peak at 1718 cm^–1^ was attributed to the C=O stretching in SBMA.^28,29^ In addition, the spectra of GSDZ samples showed the phenolic C–O vibration bands for catechol at 1288 cm^–1^, which was characteristic for phenyl ether from dopamine.^30^ The aromatic ring C=C vibration bands were overlapped with the amide bands, which were characteristic of dopamine polymerization.^21^ The amide II band typically represents N–H deformation and C–N stretching, and the amide II peak clearly shifted from 1554 to 1525 cm^–1^ for the GSDZ hydrogel samples. Higher wavenumbers in this band indicate more helical structures. The shift to lower wavenumbers indicated the tendency of the hydrogel structure to change to a random coil structure.^31^ C–H bending peak shifted from 1456 to 1449 cm^–1^, which may be resulted from the increased bond length as well as the complexation between the Zn ions and the functional groups of the polymer.^32^ The reduced intensity of the amide III peak located at 1242 cm^–1^ also indicated a loss of helical structures.^31^ In addition, the spectra of GSDZ5, GSDZ10, and GSDZ20 were similar, indicating that the concentration of Zn ions did not have a major influence on other chemical compositions in the hydrogels. The molecular weight of the complex of SBMA and dopamine determined by GPC can be found in Figure S1, indicating that the molecular weight of the oligomer varied from ∼881 to ∼1094; therefore, the addition of zinc sulfate did not show a significant impact on the synthesis process.

### 3.3 Hydrogel Morphology

**Figure 3:**
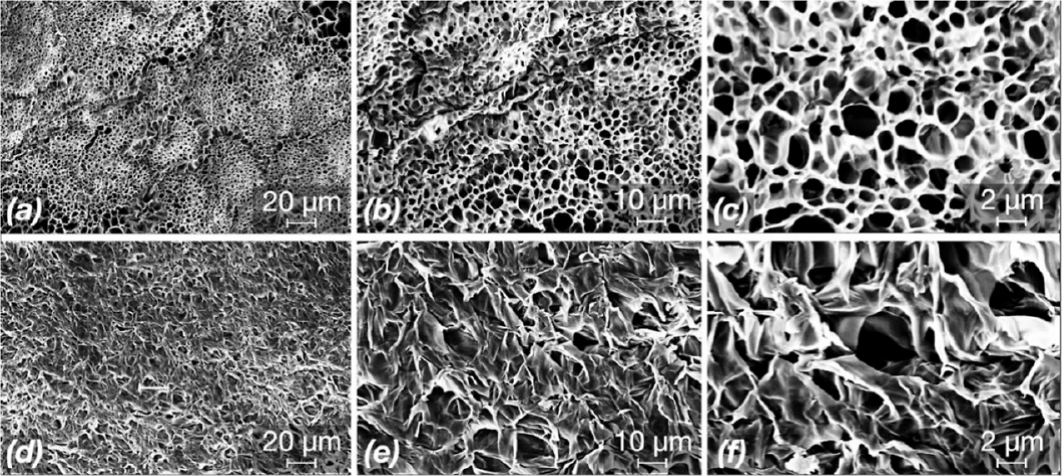
SEM images of gelatin (a–c) and GSDZ10 (d–f) hydrogel samples captured at different magnifications (scale bars: 1 μm, 10 and 2 μm).

Hydrogels and hydrogel coatings for biomedical applications are usually used in an environment containing water and microorganisms, which could lead to swelling of the hydrogel. Therefore, all specimens were immersed in DI water for 12 h to reach a swollen state before SEM scanning. As shown in Figure 3, the gelatin hydrogels had macroporous structures with pore radii varying from 0.5 to 3 μm. In contrast, GSDZ10 hydrogel samples had a denser network with fewer pores, which could be attributed to the electrostatic interactions between the metal ion and positively charged groups within SBMA. It should also be noted that the observed pores might not be a real feature of the gels since ice crystal formation during the freeze-drying could have an influence on the morphology.

### 3.4 Mechanical Properties

**Figure 4:**
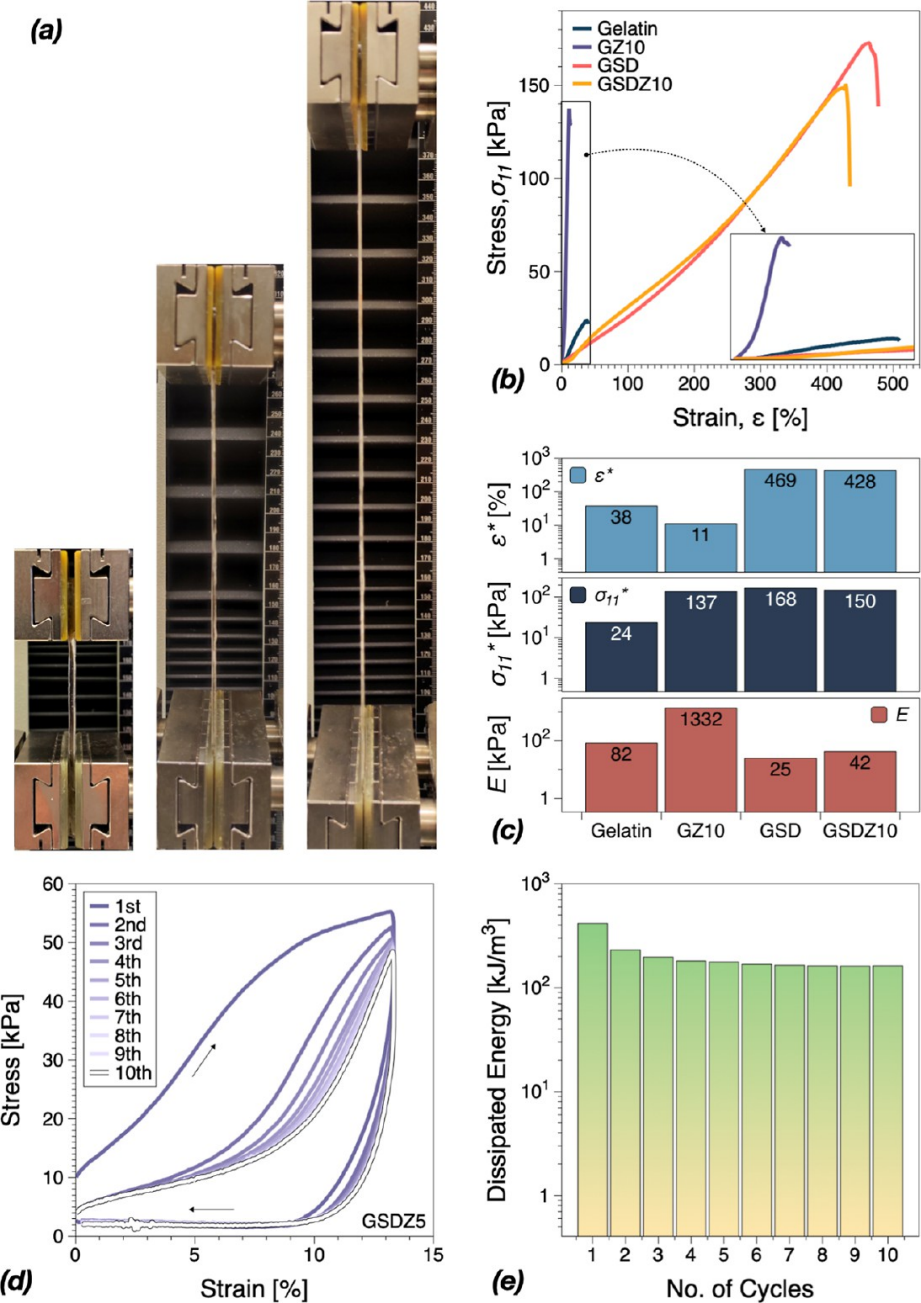
(a) Images of GSDZ10 hydrogel samples during tensile tests. (b) Tensile stress–strain behavior of gelatin, GZ10, GSD, and GSDZ10 hydrogel samples. (c) Summary of ultimate strain (ε*), stress (σ_11_*), and Young’s modulus (*E*) based on Figure S2 in the Supporting Information. (d) Consecutive loading–unloading cycles of GSDZ5 hydrogels. (e) Calculated dissipated energies.

Traditional hydrogels with a simple internal structure lack strength and have poor stretchability, which largely limits their applications. In contrast, the obtained GSDZ hydrogels had improved mechanical properties and could be significantly stretched, Figure 4a. Tensile tests were performed to investigate the ductility and toughness of the GSDZ hydrogels. As shown in Figure 4b, the pure gelatin hydrogel had very limited mechanical performance, with a stress at break of ∼24 kPa and strain at break ∼38%. The addition of the zinc sulfate (GZ10) significantly increased the stress at break and Young’s modulus, which could be attributed to the Hofmeister effect between gelatin and zinc sulfate, that is expected to induced chain bundling and hydrophobic interaction domains.^20^ However, it would also cause microphase separation resulted from the “salting-out” effect^20^ (Hoffmeister series), therefore cracks were easily grown (not shown) during the whole measurement. When the content of zinc sulfate is low, the Hofmeister effect on gelatin is very limited, which explains why the mechanical properties of GZ5 were similar to gelatin hydrogels (Figure S3). The oligomers of SBMA and dopamine led to the enhancement in tensile properties of GSD hydrogels. When the oligomers existed in the hydrogel system, the effect of zinc sulfate could vary depending on its content. In Figure S3, these results are compared to GSD samples. GSDZ5 showed much higher Young’s modulus in the beginning of the tensile tests, which was also observed on GZ10 samples. While the content of zinc sulfate was increased, Young’s modulus of GSDZ10 and GSDZ20 tended to decrease sharply. Especially when the content of zinc sulfate was very high, GSDZ20 samples exhibited low stress at break (∼40 kPa). In addition, the effect of the SBMA content on mechanical properties was investigated. In Figure S4, SBMA did not show an obvious influence on Young’s modulus while the addition of SBMA led to a higher strain at break, which could be due to more cross-links formed in the hydrogel network.

The oligomer of dopamine and SBMA provided the attainment of effective energy dissipation via multiple dynamic interactions such as electrostatic interactions, hydrophobic effects, and hydrogen bonding. However, when the concentration of zinc sulfate solution was increased to 10%, there was only a slight decrease on the breaking strain and breaking stress compared to GSD hydrogels, indicating that the oligomers of SBMA and dopamine could prevent the further expansion of microphase separation regions which happened in GZ10. Meanwhile in Figure 4c, the low Young’s modulus and the high breaking strain on GSD and GSDZ hydrogel indicated that the oligomers of dopamine and SBMA could increase the interactions between chains and the flexibility of chains, compared to gelatin hydrogel samples.

In addition to poor mechanical properties, fatigue is also regarded as one of the undesirable properties, during the use of hydrogel materials. Although hydrogels are widely used in the applications of personal care, medicine, and engineering, fatigue can occur under prolonged loading and prevent their further use. To investigate their fatigue behavior, cyclic stretching tests were performed to determine the stress–strain hysteresis. Figure 4d shows 10 successive cyclic loading–unloading tensile curves without rest between two consecutive cyclic tests exemplified on GSDZ5 hydrogels. Accordingly, the dissipated energy of each hysteresis loop is presented in Figure 4e. After the first loading cycle, a steady state of cyclic stress–stretch curves was already nearly reached, and the hysteresis loops from the second to the 10th loading were almost identical. Thus, as the orientation of polymer chains started to follow the stretching direction, this could cause additional energy dissipation. This steady state might result from the continuous breaking and reforming of dynamic bonds in the hydrogel networks, such as hydrogen bonds, ionic bonds, and hydrophobic interactions being optimized conformationally (orientation) for the type of load applied. Finally, the dissipated energies remained ∼162 kJ/m^3^ without obvious decrease, indicating that there was no macroscopic fracture in the polymer network.

### 3.5 Adhesion Properties

**Figure 5:**
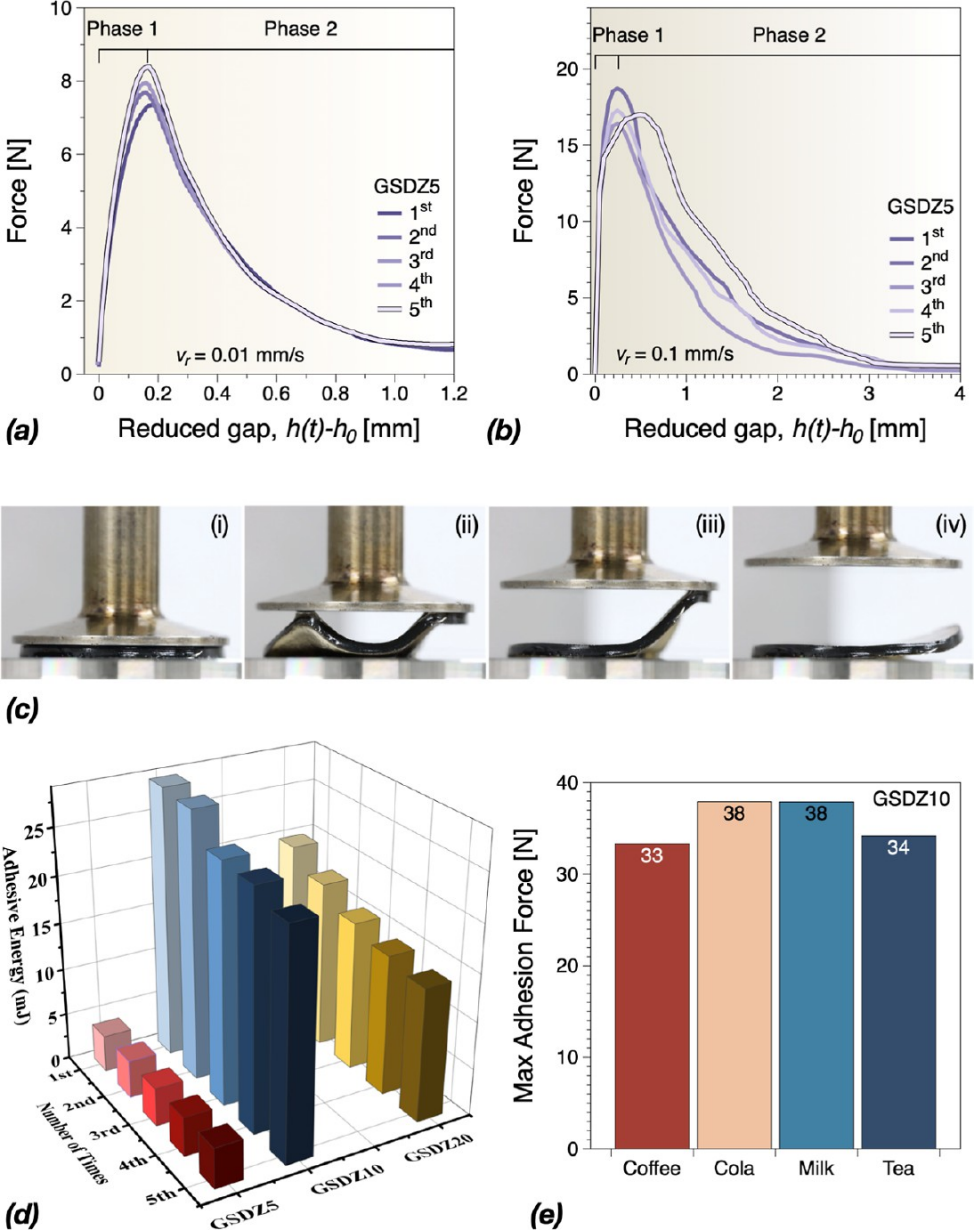
Mean force-retraction displacement curves at retraction speeds of (a) 0.01 and (b) 0.1 mm/s obtained for GSDZ5 samples. Further data can be found in the Supporting Information. (c) Photos showing the process of GSDZ hydrogels stretching and detaching during RheoTack measurements at *v*_r_ = 0.01 mm/s. (d) Evolution of calculated adhesive energies of GSDZ5, GSDZ10, and GSDZ20 during cyclical adhesion tests. (e) Maximum adhesion force of GSDZ10 hydrogels after being dipped into several types of drinks.

Many biomedical applications require hydrogels to be attached to other materials/substrates, in the form of coatings, wound dressings, strain sensors, etc. However, the abundance of water in hydrogels poses a challenge: water molecules inside hydrogels carry negligible loads and lead to poor adhesion to other materials. In nature, marine mussels can tightly attach to foreign surfaces in seawater via secreting adhesive proteins which contains DOPA, tyrosine, phenylalanine as well as cationic, anionic, and noncharged groups.^14^ Particularly, mussel foot protein-5 (mfp-5) contains the highest amount of DOPA, a functional residue that leads to strong wet adhesion, and is regarded as the most important adhesive-primer at the interface.^33^ Therefore, dopamine, a famous DOPA-derivative known as one of the most abundant neurotransmitters in the human brain, was introduced into GSDZ hydrogel coatings to improve the adhesive properties. As shown in Figure 5a,b, the force displacement of GSDZ5 hydrogels at different retraction speeds during the (rheo-)tack tests were observed, and the process can be divided into the two phases: (A) stretching and fibrillation phase and (B) detaching phase. To test the long-term adhesive properties of the hydrogels, tack tests were performed successively on the same samples. As the number of measurements increased, all the samples showed enhanced adhesion forces without any damage on the hydrogels. For example, at a retraction speed of 0.01 mm/s, the maximal adhesion force increased from 7.3 N (1st) to 8.4 N (5th), which could be the reason behind improved wettability of the substrate leading to the rapid formation of dynamic bonds between the geometry and the hydrogel. Prior to reaching the maximal adhesion force, smooth force-retraction displacement curves during stretching and debonding phase were recorded, which means that deformation took place in a uniform manner of the GSDZ5 hydrogels followed by a clean adhesive failure without evidence of cohesive failure or fibrillation. When the adhesion was beyond the maximum force, bonds started to break arbitrarily, and the force was more randomly scattered during the detaching phase. This was more obvious when a higher retraction speed was applied. In addition, with increasing retraction speed, the maximal adhesion force was also enhanced because of material’s viscoelastic nature which means that the time scale of the deformation significantly exceeds the relaxation time of the hydrogels.^34^ However, the displacement of the maximal adhesion force (∼1.45 mm) was hardly dependent on the retraction speed, indicating that the detaching displacement is a strain-triggered parameter. Meanwhile GSDZ hydrogels could be attached to various types of surfaces (Figure S5), such as paper, skin, wood, plastic, rubber, and steel. In general, the adhesion of GSDZ hydrogels and different substrates can be achieved through supramolecular interactions.^35^ For example, the hydrogen bonds formed between catechol groups of dopamine and biological materials; glass and hydrogels generated electrostatic interactions; Through the coordination interaction, the carboxyl group may attach hydrogels to the metal. Interestingly, the increase of zinc sulfate could also influence the adhesion energy and the maximal adhesion force, as shown in Figure 5d. The adhesion energy was increased from ∼4.07 to ∼26.27 mJ and the maximal adhesion force was increased from ∼17.26 to ∼31.54 N when the concentration of ZnSO_4_ was increased from 5 to 10% at the retraction speed of 0.1 mm/s (Figure S6). Among all the tested samples, GSDZ10 hydrogel exhibited the highest adhesive energy. Moreover, it is suggested that SBMA segments could form a dense hydration layer as protection after absorbing large amount of water molecules through ionic solvation.^36^ Therefore, SBMA was introduced and took effect with other components in order to improve the service life of GSDZ hydrogels in daily environments. As shown in Figures 5e and S8, the GSDZ10 hydrogels could still retain high adhesion force after being contaminated by different drinks, which indicated promising antifouling properties. In addition, it is observed that the content of SBMA could influence the adhesive force. In Figure S9, without the addition of SBMA, GS0DZ5 exhibited the lowest adhesive force among all of the tested samples. While after the SBMA was introduced into the hydrogel, the adhesive forces of GS1DZ5 and GS2DZ5 significantly increased, which could be the consequence of the increased ionic bonds and hydrogen bonds. GS2DZ5 showed the maximal force (−33 N), which was the highest among all tested samples. Due to the hydrophilicity of SBMA, the further increase in the content of SBMA could lead to more “free” water molecules at interface which prevent interfacial physical interactions, therefore compared to GS2DZ5, the adhesive force of GSDZ5 was lower. The effect of temperature on adhesion was also investigated. As shown in Figure S10, when the temperature decreased from 25 to 15 °C, the maximal adhesive force increased from −14 to −31 N, which could be due to the enhancement in the hydrogen bond at low temperature. It should be noted that when the sample detaches and fibrillates, air of different temperature also sneaks in, leading to the temperature gradient on the hydrogels.

### 3.6 Self-Healing Properties

**Figure 6:**
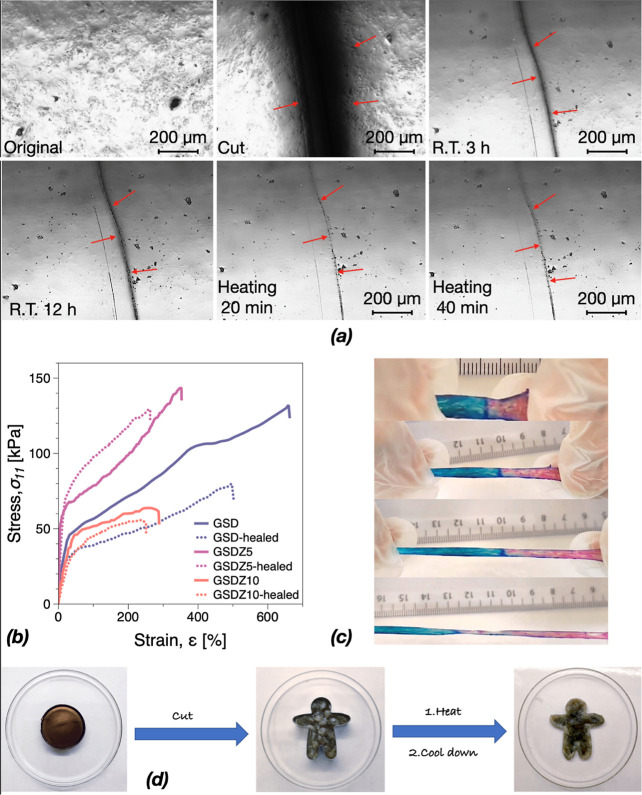
(a) Microscopy images showing the process of self-healing on GSDZ5 hydrogel samples. (b) Tensile stress–strain curves of original and healed GSD, GSDZ5, and GSDZ10 hydrogel samples. (c) Photos showing the healed GSDZ5 hydrogel samples being stretched. (d) Photos showing that the GSDZ hydrogels can be remolded.

Hydrogels are subjected to external mechanical forces during their use, which may destroy the integrity of the hydrogel network to a certain extent and further influence the performance of the hydrogel itself. Therefore, in the field of biomedical engineering, sometimes there is a need to design a hydrogel with effective and autonomous self-healing ability for quickly recovering their structural integrity and providing sustained antibacterial ability. Due to the reversible nature of dynamical physical cross-linking in the hydrogel network, such as hydrogen bonds and coordination bonds, the obtained hydrogel samples showed excellent self-healing properties. To be specific, the concept of quick equilibrium between the reversible dissociation and recombination of the dynamic connections serves as the foundation for self-healing hydrogels.^2^ This suggests that in order to build the gel network, functional groups on the gelatin and the oligomer chains, such as hydroxyl, carboxyl, and amino groups, are required. To fill and link the injured region and repair the hydrogels, certain “mobile phases” are formed in or near the damaged area when the hydrogels are damaged. After the initial damage (cutting), all hydrogels could heal themselves at room temperature without external stimulation, and this self-healing could be further improved by heat, which is exemplified through microscopy images on GSDZ5 in Figure 6a. After the third hour of healing, the lesion of the sample had been closed and under heat treatment at 60 °C, it was further repaired by the sol–gel transition, which could be a potential to combine with low-temperature sterilization, for example pasteurization, in the application of microcapsules.^37^ At this point, the repaired specimen could already be stretched and would not break at the cut as exemplified on GSDZ5 in Figure 6c. Tensile tests were also performed on the original and healed GSD and GSDZ samples to quantitatively analyze the self-healing properties of the hydrogels. As can be seen in Figure 6b, all of GSD, GSDZ5, and GSDZ10 samples exhibited good mechanical properties. Compared to the original ones, the strain and stress at break of all the healed samples decreased, indicating that there were damages in the network that could not be recovered fully. Moreover, as shown in Figure 6d, the chopped hydrogel samples with the addition of little water could be reshaped and be healed after heating and cooling due to the sol–gel transition of gelatin.

**Figure 7:**
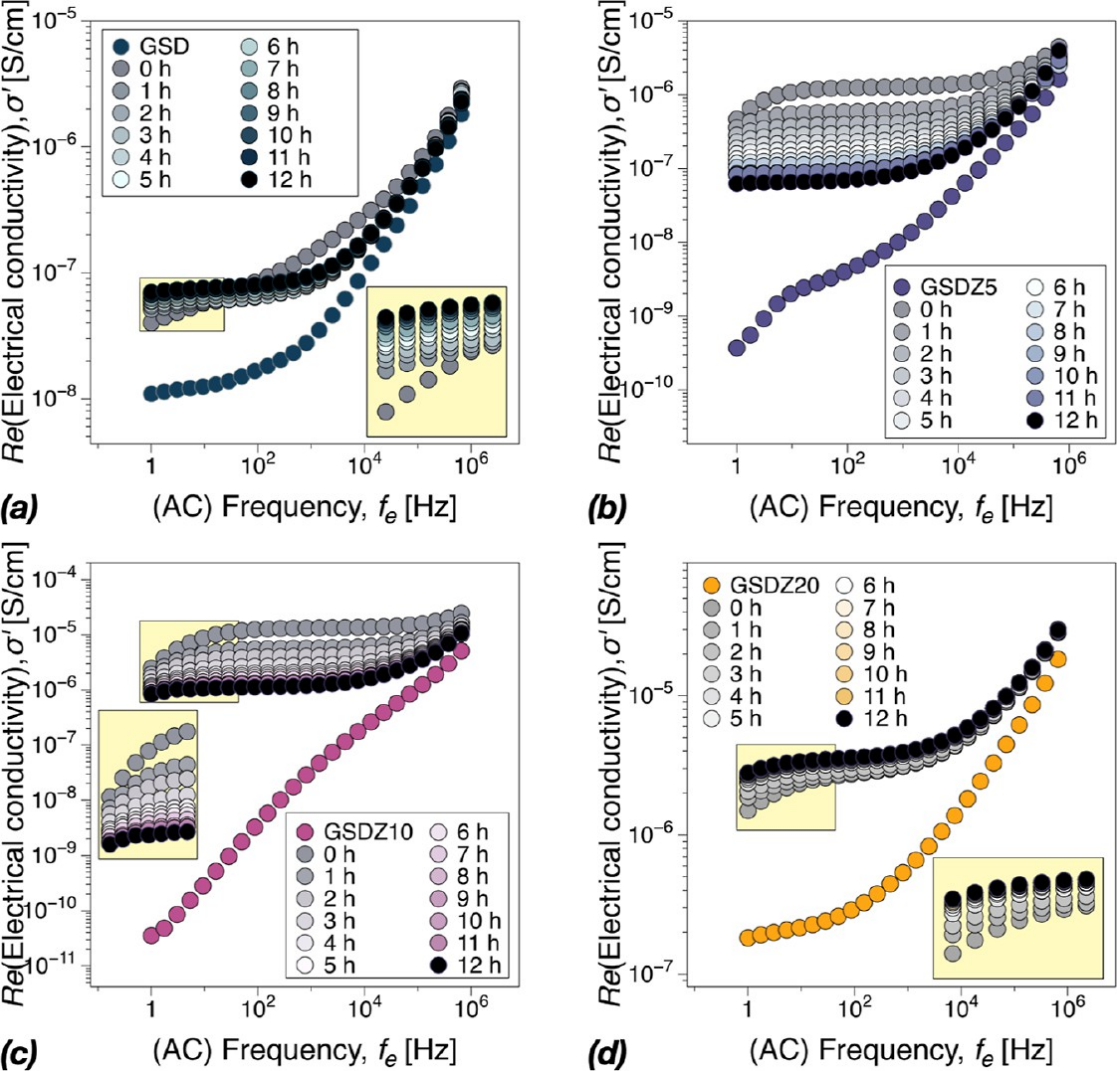
Self-healing characterized through dielectric spectroscopy for (a) GSD, (b) GSDZ5, (c) GSDZ10, and (d) GSDZ20.

Dielectric spectra determined in situ during the self-healing process are shown in Figure 7. In general terms, factors that affect the mobility of ions in hydrogels, such as the amount of free (unbound) water, are crucial.^38^ It is important to note that in addition to the σ_DC_ conductivity plateau in the limit of low frequencies, eq 1, for ionic conductive materials, a further decrease in conductivity is expected at even lower frequencies. This has been associated with electrode polarization, i.e., the blocking of ions at the sample-electrode interface. In the following, we interpret the data in terms of σ_DC_ conductivity plateau, whether in the low- or intermediate-frequency region. Due to the long time scales associated with self-healing, a wet GSDZ5 sample was used to investigate drying dynamics within 12 h (Figure S11). The results showed a decrease of 5.2 × 10^–5^ to 3.5 × 10^–5^ S/cm (38%) in σ_DC_. This limited reduction was expected due to the small sample surface area in contact with the ambient air during testing. Overall, the pristine hydrogels (before cutting) highlight the typical variable initial conditions possible in such systems in terms of the water content. However, after cutting the samples with a wet blade, the conductivity exhibited a significant increase due to the increase in ion mobility. Thus, the self-healing process was accompanied by σ_DC_ generally proportional to the zinc sulfate content. Notably, after 12 h, GSD and GSDZ5 settled for similar σ_DC_, while for GSDZ20, the conductivity continued to increase during the healing process. Additional tests have shown an increase in σ_DC_ with the increasing SBMA content while keeping the zinc sulfate concentration constant, see Figures S11–S13. Thus, the similarity between GSD and GSDZ5 after cutting suggests that the conduction mechanism in GSDZ5 remains dominated by the SBMA content, which is the same for both samples. The increase in σ_DC_ during self-healing for GSDZ20 could be attributed to possible phase separation when the zinc sulfate concentration is high. While the data could be subject to further analysis, we can conclude that the healing process could be successfully monitored using the setup.

### 3.7 Sensing Properties

**Figure 8:**
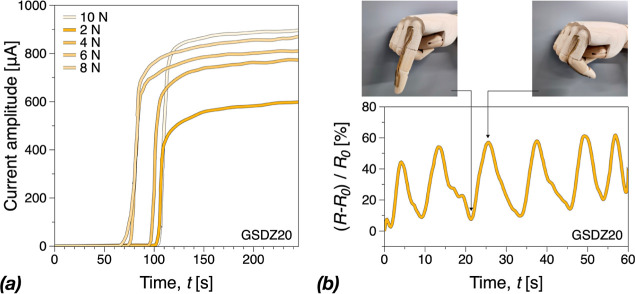
(a) Current amplitude during a rheo-dielectric test of GSDZ20 hydrogel samples applied by external forces in real-time; and (b) resistance variations of the GSDZ20 wearable sensor as a function of time when fingers moved.

With the highest electrical conductivity, GSDZ20 hydrogels could be used as flexible wearable devices, aimed to achieve the real-time monitoring of organ function in human, personalized diagnosis and treatment. We briefly note that based on the data in Figure 8 and discussion thereof, the conductivity of the hydrogels is significantly affected by their water contents and therefore lower concentrations of zinc sulfate variants could also be tuned for sensing. Furthermore, this dependence on the moisture content could be exploited further for diagnostics. Figure 8a shows the current amplitude of the GSDZ20 hydrogel samples applied by external forces in real-time. Generally, the current amplitude of GSDZ20 increased as the external force increase. This is expected, as changes in contact area and sample height at the same time with the application of the force. Thus, as the force increases, the increase in conductivity is comparatively lower due to limits in the compressibility of the samples. Based on the strain sensitivity and mechanical and adhesive properties, the GSDZ20 hydrogel could be attached on human fingers and used as a soft sensor device to monitor finger motions, Figure 8b. It can be seen that the resistance of GSDZ20 hydrogel increased as the sample was bent, and the peaks appeared when the finger was bent to 90°.

### 3.8 Antifreeze Properties

**Figure 9:**
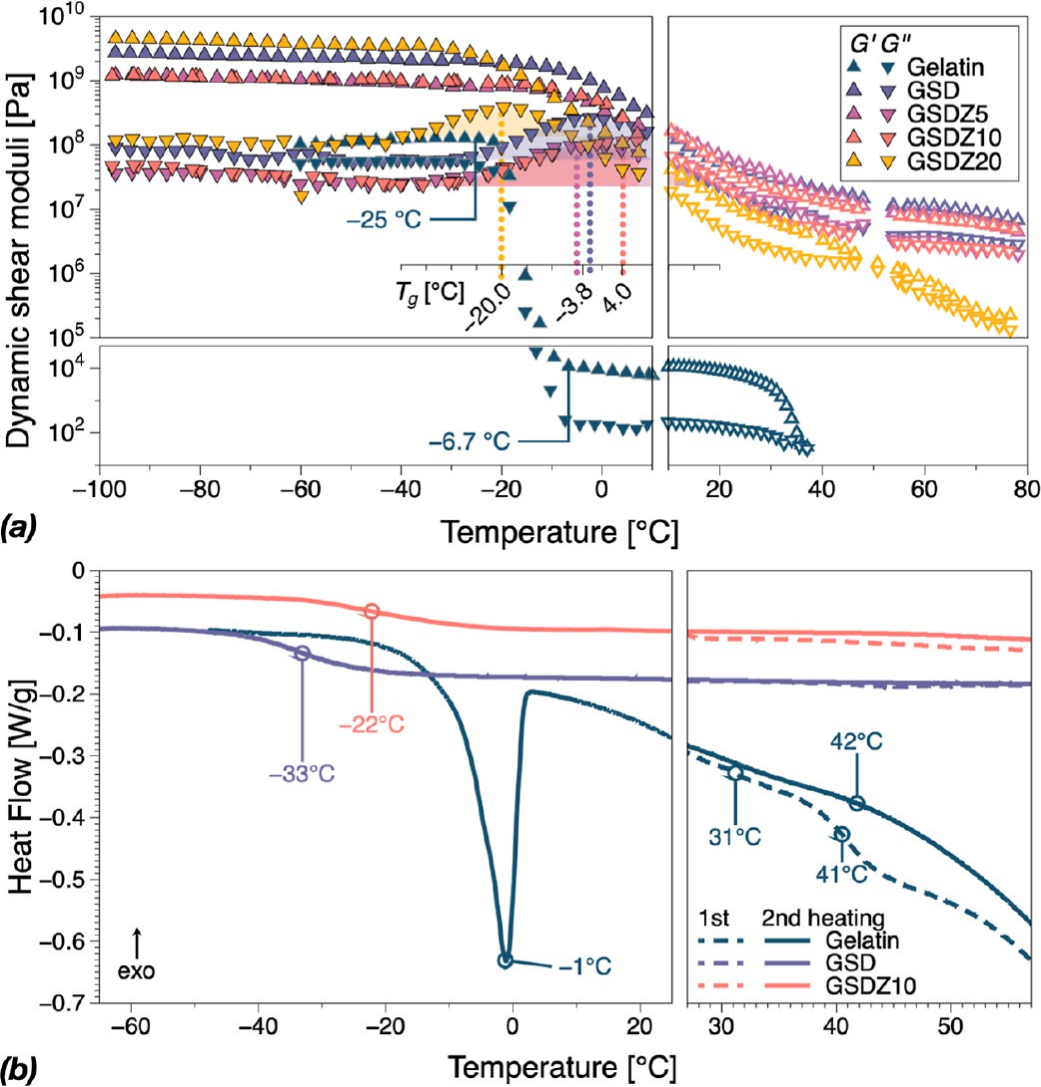
(a) Shear dynamic moduli, *G*′, *G*″, in the (−100,80) °C temperature range comprising two separate sets of tests (−100,10) °C and (10,80) °C corresponding to antifreeze and thermal stability tests. The areas filled under the curves illustrate the broadness of the *T*_g_ region based on the peak width in *G*″. (b) DSC curves of gelatin and GSD and GSDZ10 hydrogels in a similar temperature range.

Dynamic moduli data from the DMTA moduli across the entire temperature range are listed in Figure 9a. The low-temperature behavior is discussed here in the context of antifreeze properties, whereas from room temperature to the higher end, the results are discussed in terms of thermal stability in the following section. Similarly, the corresponding DSC curves are presented in Figure 9b.

Freezing is often regarded as an unfavorable factor to the structural integrity and performance of materials. As can be observed in Figure 9a, for pristine gelatin with decreasing temperature, the dynamic moduli increased sharply by around 5 orders of magnitude between −6 and −25 °C. The second heating thermogram presented in Figure 9b indicates a clear melting peak at −1 °C, range that corresponds very well to the abrupt changes in gelatin mechanical properties. In contrast, GSD-based hydrogels experienced only a glass-transition temperature, *T*_g_, in the range of −4,4 °C (based on the peak in *G*″), with the exception of GSDZ20. Glass transitions for GSD and GSDZ10 were also observed at DSC thermograms in Figure 9b at −33 and −22 °C, respectively. Transitions cover quite a wide range of temperatures similar to mechanical characterization data in Figure 9a, however are shifted toward lower temperatures. This can be attributed to the difference in the testing procedures, particularly, to heating/cooling rates, water content and sample sizes, that inevitably affect heat transfer. The antifreeze performance even in the absence of zinc sulfate could be due to the increase in content of hydrophilic functional groups on SBMA-dopamine oligomers and the number of hydrogen bonds and electrostatic interactions formed with free water. This phenomenon is known to disrupt the ordered arrangement of water molecules and to enhance in the freezing resistance of the hydrogels^39^ since compared to the interactions between water molecules, energy-wise, this is more favorable.^40^ Besides that, the number of oligomers as additives in the hydrogel found to be highly increased compared to pure gelatin, so the colligative properties could contribute to the antifreeze properties of GSD hydrogels,^41^ which means that it depends on the quantity of particles, not on their chemical properties. To be specific, high solute concentrations are thought to make the creation of large ice nuclei more difficult, which lowers the chance of freezing. This is because there is less free water in the solution.^40^ While the concentration of zinc sulfate had no significant effect below 20%, GSDZ20 showed a clear shift of the *T*_g_ toward lower temperatures, i.e., ca. −20 °C. In addition, the effect of the SBMA content on antifreeze properties was investigated. As shown in Figure S14, no freezing/melting peak was found below 0 °C and as the content of SBMA was increased, the *T*_g_ was shifted from 17 °C in GS0DZ5 to −2.3 °C in GS2DZ5.

### 3.9 Thermal Stability

Dynamic mechanical and DSC data shown in Figure 9 were considered in relation to the thermal stability of the hydrogels. With increasing temperature, the dynamic moduli of gelatin dropped dramatically when the temperature reached around 37 °C, accompanied by a crossing of the dynamic moduli, as it transitions from a gel to solution.^3^ From DSC data, there is an indication of the presence of the expected two transitions as gelatin passes through the sol–gel transitions at 31 and 41 °C.^42^ As Djabourov and Papon reported,^43^ these two peaks indicate that the hydrogen bonds in the gelatin network were first destructed at around 30 °C and the further heating caused the conformation change of partly reserved triple-helix structures, resulting in an additional transition in DSC curve of gelatin hydrogel. During the second heating, it can be seen that these two transitions spread out into one first-order transition with deflection point at 42 °C.^44^ In contrast, for GSD and GSDZ5 and GSD10 hydrogels, there was a clear elastic-dominated material response, *G*′ > *G*″ over the whole temperature range. This behavior, characteristic to cross-linked systems indicates the integrity of the polymer network and its thermal stability, which could be due to the enhancement in supramolecular interactions in the hydrogels, such as the coordination bonds among the GSDZ hydrogel network.^45^ No melting peaks appeared during the second heating of GSD or GSDZ10.

Furthermore, TGA was adopted to evaluate the degradation behavior of the obtained hydrogels. As shown in Figure S15, two distinct transitions are visible for gelatin with inflection points at 64 and 110 °C, where around 70% weight of the sample is lost. The first one lasts from the starting temperature of 30 °C up to ∼100 °C and corresponds to the water evaporation process, while the second one lasts until 276 °C and indicates the initiation of the degradation process.^46^ Further degradation stage starts at 276 °C. In contrast, for GSDZ10, the first stage started shortly after the experiment started (43 °C) and lasted until 200 °C. It was accompanied by a small mass reduction (∼5%), which could be due to water evaporation and the presence of the tightly bound water resulted from the hydrophilicity of zwitterionic SBMA.^47^ The second stage, with significant mass drop of 48% began at 221 °C and lasted until ∼440 °C, indicating major structural changes in the hydrogel network, such as the degradation of gelatin, dopamine, and SBMA. As the temperature increased to ∼460 °C, a third transition could be identified, which could be attributed to the initial decomposition of ZnSO_4_ and further transformation to ZnO·2ZnSO_4_ at higher temperatures.^48^

### 3.10 Rheology of Precursor Solutions and Topography of Coatings

**Figure 10:**
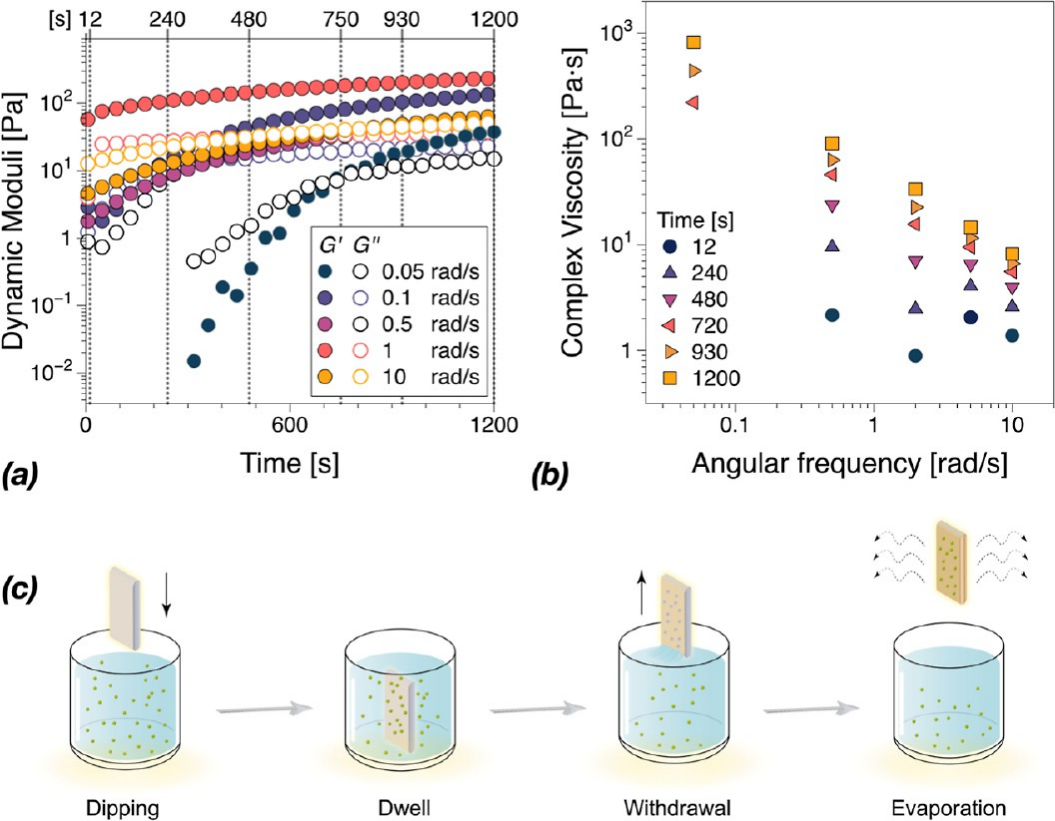
(a) Dynamic time sweep at several angular frequencies with a constant strain amplitude in the linear viscoelastic regime on the GSDZ10 hydrogel precursor solution. (b) Complex viscosity magnitude as a function of the angular frequency from isochronous data in figure (a). The corresponding dynamic moduli can be found in Figure S16. (c) Illustration showing the process of coating fabrication via the technique of dip coating.

In addition, the rheological properties of the precursor solution were investigated to assess their gelation behavior and explore the possibility of using them for coating applications. In the precursor solutions, there are different interactions and cross-linking ongoing between components, and the rheological properties of precursor solutions are highly time dependent. Therefore, a dynamic time sweep at several angular frequencies with a constant strain amplitude is chosen. As shown in Figure 10a, the shear dynamic moduli of GSDZ10 precursor solution varied from 10^–2^ to 10^2^ Pa in the beginning and continuously increased as the measuring time was prolonged due to the cross-linking of the hydrogel. Meanwhile, the sol–gel transition derived from gelatin was observed within 20 min under different angular frequencies. To properly assess the formation of a gel, the rheological conditions of equal dynamic moduli over the whole test frequency range need to be verified. Therefore, in Figure 10b, isochronous data were extracted from the time-dependent data in Figure 10a at selected time steps and converted into frequency-dependent data. While in Figure 10b, the magnitude of the complex viscosity is represented, the angular frequency dynamic moduli are reported in Figure S16. Thus, it can be observed that the viscosity functions suggest a zero-shear Newtonian plateau followed by a shear thinning region, as typical of polymeric solutions, up to ca. 480 s. As can be seen in Figure S16, 480–750 s is the gel point region, with *G*′ ≈ *G*″ over the entire frequency range studied. Above this time range, the complex viscosity magnitude functions showed a power-law behavior and *G*′ > *G*″ indicated the formation of a cross-linked gel.

Shear thinning is one of the important factors in dip-coating, Figure 10c, as the viscosity is reduced in the vicinity of the substrate during the dipping and withdrawal phase.^49^ Once the substrate is withdrawn, the rapid sol–gel transition and high viscosity could help the precursor solution remain firmly attached to the surface without breaking the structure of the solution layer, which could lead to highly uniform coatings after evaporation. In addition, atomic force microscopy (AFM) was used to test the topography and roughness of the GSDZ hydrogel coatings. As shown in Figure S17a, surface features of bare glass cover surface were generally flat and smooth, with RMS roughness values of 0.18 nm. After the hydrogels were coated, differences in the surface morphology were observed, with typical polymer aggregates appearing on the surface. However, in Figure S17b, the surface roughness (0.20 nm) did not change significantly when the glass was coated with GSDZ hydrogel, indicating that the gelatin-based hydrogel coatings did not dramatically change the surface roughness of the substrates. In addition, the increase of zinc sulfate had no effect on the morphology and roughness of the coating itself.

### 3.11 Antibacterial Properties

**Figure 11:**
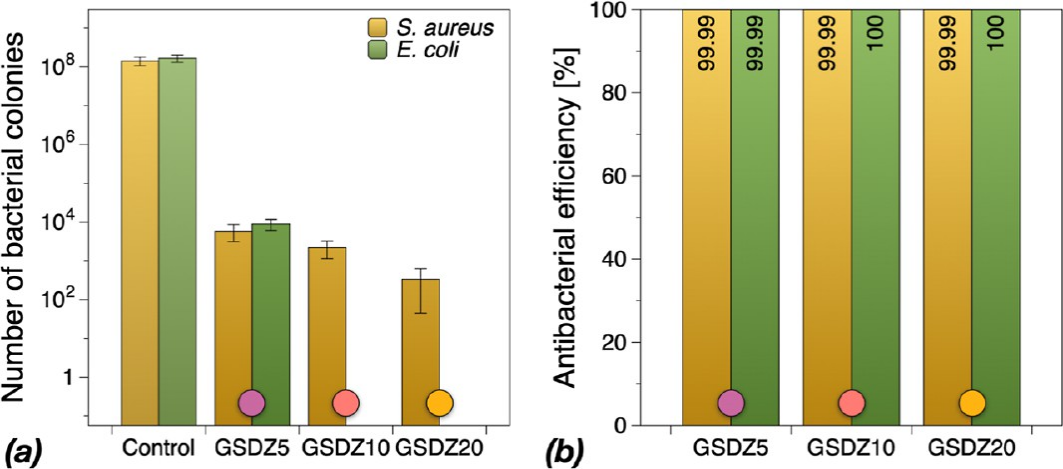
Comparison of antibacterial activities of control samples, GSDZ5, GSDZ10, and GSDZ20 hydrogel coatings against *S. aureus* and *E. coli* (a) and corresponding antibacterial efficiency (b).

Ideal biomedical surfaces are expected to prevent the biofilm formation either by preventing the bacterial adhesion or by deactivating the adhered bacterial cells. In this work, *S. aureus* and *E. coli* were used as model Gram-positive and Gram-negative bacteria, respectively, since they are common bacterial pathogens forming biofilms on medical devices and implants.^50^ Stainless steel was selected as a biomedical substrate since it is a common material to be used in medical devices. The steel discs were dip coated with GSDZ hydrogels with varying concentrations of zinc sulfate. To test the antibacterial efficiency, noncoated and hydrogel-coated discs were exposed to bacterial culture for 24 h at 37 °C. Biofilm preventive activity was measured by means of colony forming units (cfu) counting and SEM imaging. As shown in Figure 11a, the bare steel disc harbored 1.43 × 10^8^ and 1.67 × 10^8^ cfu/mL of *S. aureus* and *E. coli,* respectively. However, the steel discs with GSDZ5 hydrogel coating had only 5.83 × 10^3^ and 8.83 × 10^3^ cfu/mL of *S. aureus* and *E. coli,* respectively, demonstrating the strong antibiofilm properties of the hydrogel coatings. It can be seen that the number of viabilities of bacterial cells decreases as the concentration of zinc sulfate increases in the hydrogel samples. Surprisingly, when the concentration of zinc sulfate solution increased to 10% or more, there were no *E. coli* colonies detected suggesting the complete deactivation of *E. coli* cells. The activity against *S. aureus* was also improved with the presence of increasing concentrations of zinc sulfate. The observed antibiofilm activity against *E. coli* and *S. aureus* suggests that the developed hydrogel is more effective against Gram-negative bacterial cells compared with Gram-positive counterparts. This difference in bactericidal activity could be due to the composition of cell wall of Gram positive and Gram-negative bacterial cells. The cell wall of Gram-positive bacterial cells consists of thick layer of peptidoglycan (20–80 nm) suggested to act as a physical barrier to protect the cells from external stimuli such as metal ions or environmental assaults.^51−53^ To further quantify viability reduction, the antibacterial efficiency was calculated as relative percentage of reduction to the control, i.e.
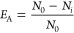
where *E*_A_ is the antibacterial efficiency, *N*_0_ is the numbers of cfu determined for the control sample, and *N*_*i*_ is the numbers of cfu determined for stainless steel coated by GSDZ hydrogel.

As shown in Figure 11b, all GSDZ hydrogel coatings showed over 99.99% antibacterial efficiencies against both Gram-positive and Gram-negative bacteria, especially for GSDZ10 and GSDZ20, which exhibited 100% antibacterial efficiency against *E. coli*. In addition, the numbers of bacterial colonies on GSDZ20 coating were around 330 for *S. aureus* and 0 for *E. coli*, which are lower than those on other hydrogel coatings; therefore, GSDZ20 exhibited the best antibacterial properties.

**Figure 12:**
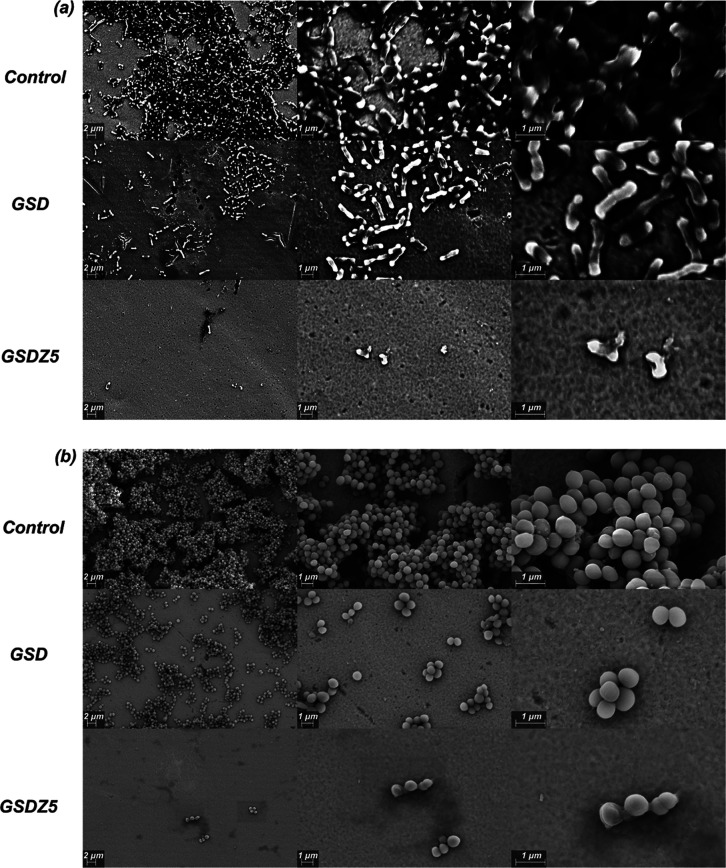
SEM images of *E. coli* (a) and *S. aureus* (b) grown on the control sample and GSD and GSDZ5 hydrogel coatings.

**Figure 13:**
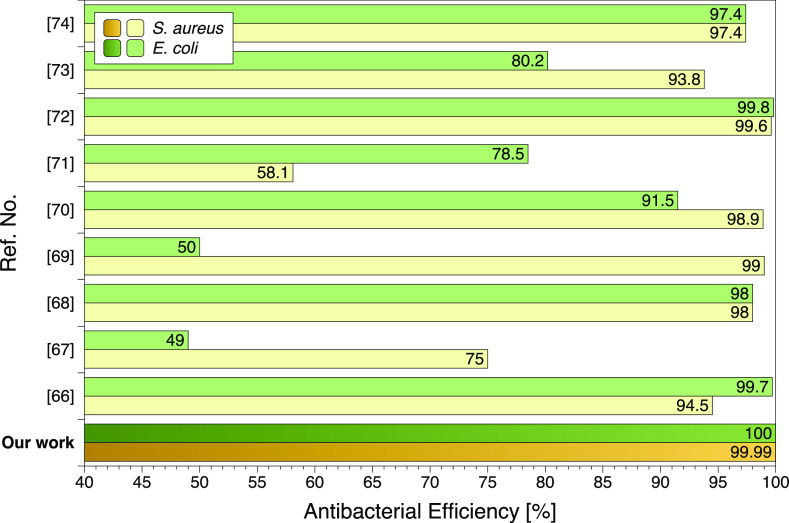
Comparison between the present study and other gelatin-based hydrogels showed similar levels of the antibacterial efficiency. Comparison between the present study and other gelatin-based hydrogels showing similar levels of antibacterial efficiency. In refs 66, 69, and 74, the antibacterial activities were tested by the colony count method; in refs 67, 71, 72, and 73, the antibacterial activities were tested by the disk diffusion method; and in refs 68 and 70, the antibacterial activities were tested by the optical density method.

Although the antibacterial efficiency of hydrogel coatings was clearly observed from viability testing based on cfu counting, it was not clear whether this reduction in viability is due to repellent or bactericidal activity of the hydrogel coatings. To examine this further, bacterial cells grown on hydrogel-coated and noncoated (stainless steel) surfaces were examined using SEM and representative images are presented in Figure 12. The SEM images show that the stainless-steel disc with GSD hydrogel coating had much lower bacteria densities of both *S. aureus* and *E. coli* compared to the uncoated control. The excellent antibiofouling properties could be the result of improved hydrophilicity and introduction of the zwitterionic SBMA.^54^ Smaller number of microcolonies and reduced density of bacterial cells in each microcolony were found on samples coated with GSD-coated hydrogels. However, the remaining bacteria had intact structures and smooth membranes, suggesting that GSD hydrogel coating may not be bactericidal but prevent the bacterial adhesion to surface. When zinc sulfate was added, GSDZ hydrogel coatings could be observed to deactivate the bacterial cells by causing severe membrane damage. This result suggests that GSDZ5 hydrogel coating not only repelled the adhesion of both Gram-positive and Gram-negative bacteria but also killed them efficiently as the SEM image captured unviable bacteria with leaked cytosol and broken membranes of the bacterial cell. It is worth noting that the levels of the antibacterial efficiency reached through the newly developed hydrogels are extremely high. Similar but not matched antibacterial performance have been reached in very few studies,^55−63^ see Figure 13.

## 4 Conclusions

In this work, we engineered a versatile gelatin-based hydrogel with strong adhesion, excellent fatigue-resistance, thermal stability, antifreeze properties, electrical conductivity, self-healing, and antibacterial properties, via the in situ synthesis of oligomers of dopamine and SBMA. Meanwhile by adjusting the content of zinc sulfate, the properties of the hydrogel materials could be tuned for further biomedical applications such as wearable sensors monitoring functions of organ and antibacterial coatings on biomedical devices. More importantly, the methods of RheoTack and rheo-dielectric were first proposed to investigate the adhesive, self-healing, and electrical properties of the hydrogel materials along with the means of cfu counting and SEM imaging, showing 99.99% and 100% antibacterial efficiency against *S. aureus* and *E. coli*, respectively, as well as antibiofouling properties. Therefore, the developed organic/inorganic hybrid gelatin-based hydrogels are promising candidates for biomedical applications.

## Supporting Information Available

GPC curves of SBMA-dopamine oligomers, stress–strain curves of gelatin, GZ5, GZ10, GSD, and GSDZ samples; force-retraction displacement curves of GSDZ samples; dielectric spectra of GSDZ samples; DSC curves of GS0DZ5, GS1DZ5, and GS2DZ5 hydrogels; TGA curves of gelatin and GSDZ10 hydrogels; dynamic moduli at several angular frequencies of GSDZ10 samples; and AFM images of GSDZ samples (PDF)
