# Supplementary material for: Biomimetic Antibacterial Gelatin Hydrogels with Multifunctional Properties for Biomedical Applications
Source: ACS Appl Mater Interfaces. 2023 Nov 17;15(47):54249–65. doi: 10.1021/acsami.3c10477 (PMC10694820; doi:10.1021/acsami.3c10477)
Supplement: Supplementary file 1 — am3c10477_si_001.pdf [file am3c10477_si_001.pdf]

Supplementary information

Biomimetic antibacterial gelatin hydrogels with multifunctional properties for biomedical applications

Hengzhi Ruan[1, *], Marko Bek[1], Santosh Pandit[2], Alexandra Aulova[1], Jian Zhang[2], Philip Bjellheim[3], Martin Lovmar[2,3], Ivan Mijakovic[2,4, **], Roland Kádár[1, **]

[1] Department of Industrial and Materials Science, Chalmers University of Technology, 412 96 Göteborg, Sweden

[2] Department of Biology and Biological Engineering, Chalmers University of Technology, 412 96 Göteborg, Sweden

[3] Wellspect Healthcare AB, 431 21 Mölndal, Sweden

[4] The Novo Nordisk Foundation Center for Biosustainability, Technical University of Denmark, 2800 Kongens Lyngby, Denmark

*Main corresponding author: hengzhi@chalmers.se

** Emails: ivan.mijakovic@chalmers.se; roland.kadar@chalmers.se

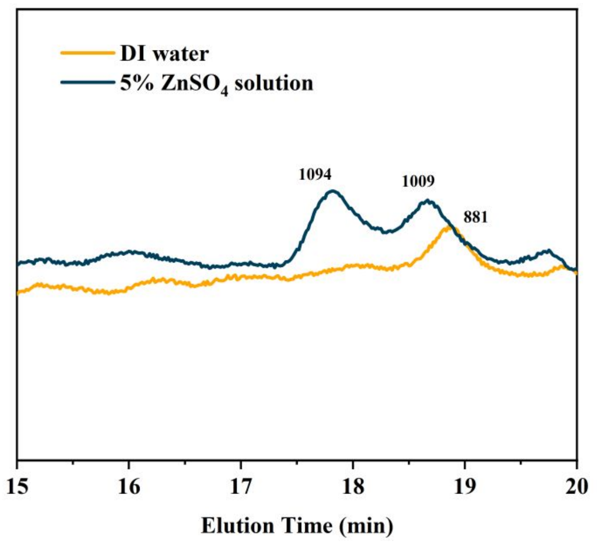

Figure S1. GPC curves of SBMA-dopamine oligomers synthesized in DI water and 5% $ZnSO_4$ solution.

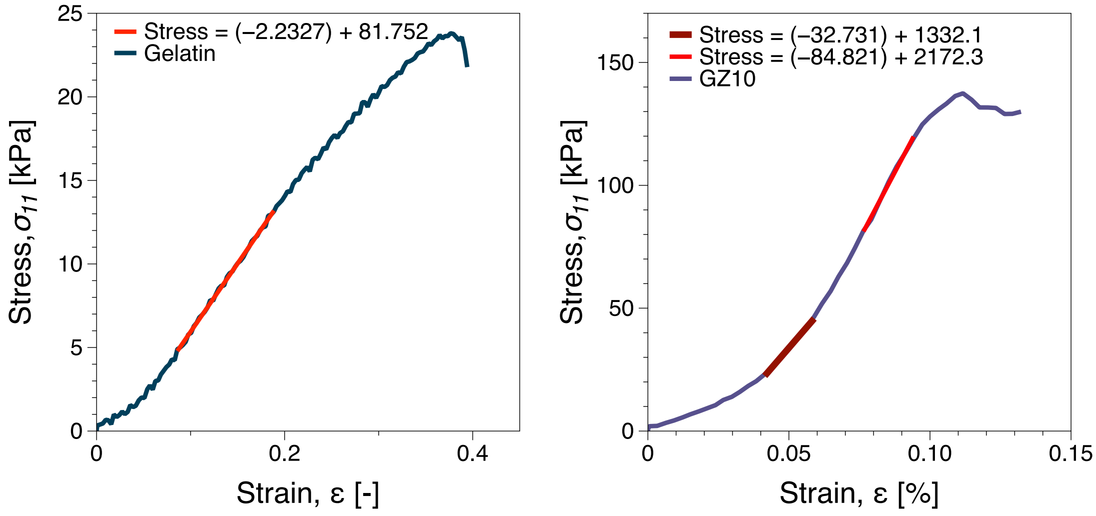

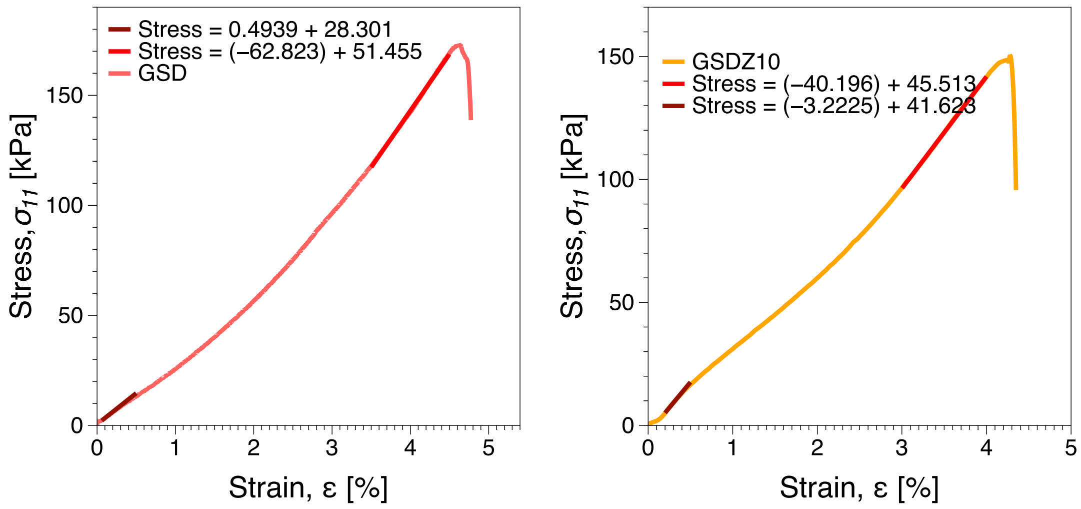

Figure S2. Stress-strain curves for each individual tests in Figure 4 together with the linear fits for obtaining Young's modulus and modulus at stiffening, where present, before failure.

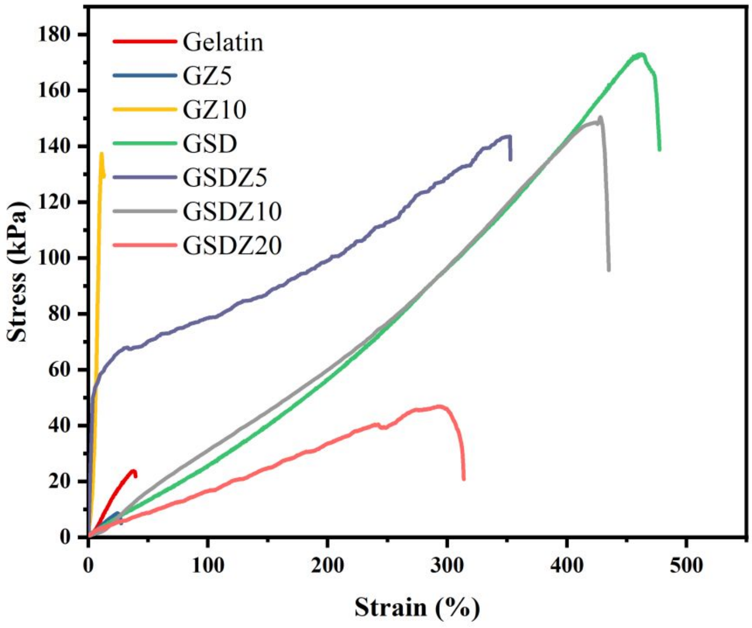

Figure S3. Tensile stress–strain behaviour of gelatin, GZ5, GZ10, GSD, GSDZ5, GSDZ10 and GSDZ20 hydrogel samples.

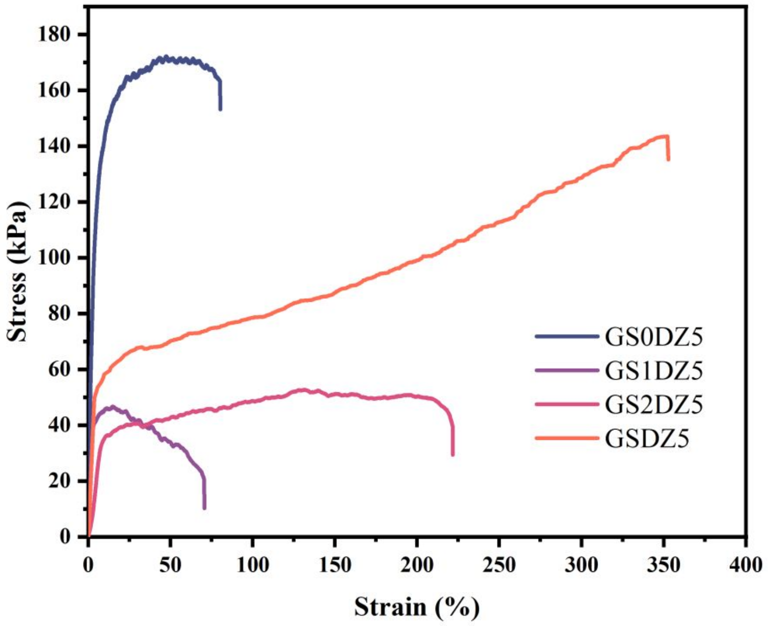

Figure S4. Tensile stress–strain behaviour of GS0DZ5, GS1DZ5, GS2DZ5 and GSDZ5 hydrogel samples.

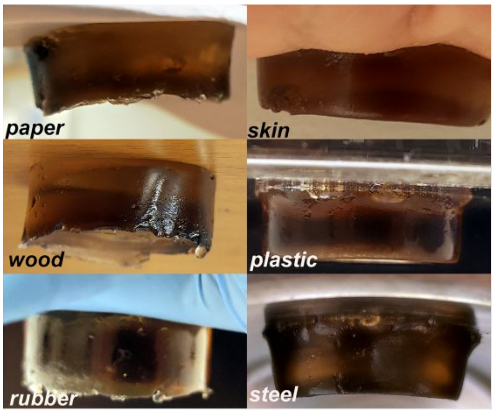

Figure S5. The photos of GSDZ hydrogels attached to different types of surfaces.

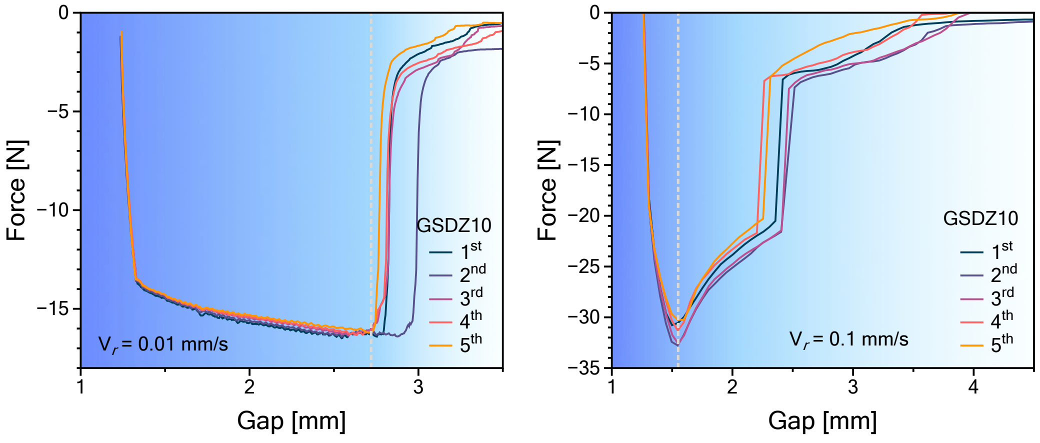

Figure S6. Force-retraction displacement curves at retraction speeds of 0.01 and 0.1 mm/s mm/s obtained for GSDZ10 samples. Note that in contrast to the data in Figure 5 where the absolute value of the force is represented, here the raw force values as recorded by the rheometer are presented.

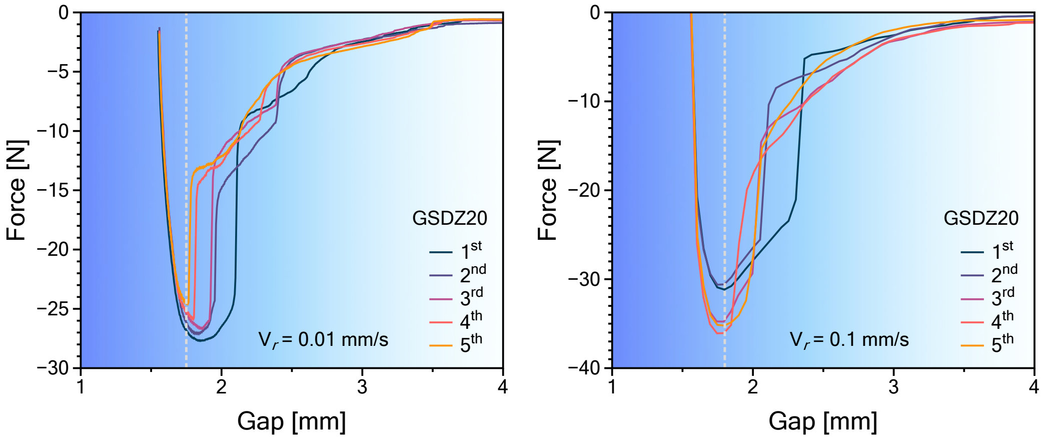

Figure S7. Force-retraction displacement curves at retraction speeds of 0.01 mm/s and 0.1 mm/s obtained for GSDZ20 samples. Note that in contrast to the data in Figure 5 where the absolute value of the force is represented, here the raw force values as recorded by the rheometer are presented.

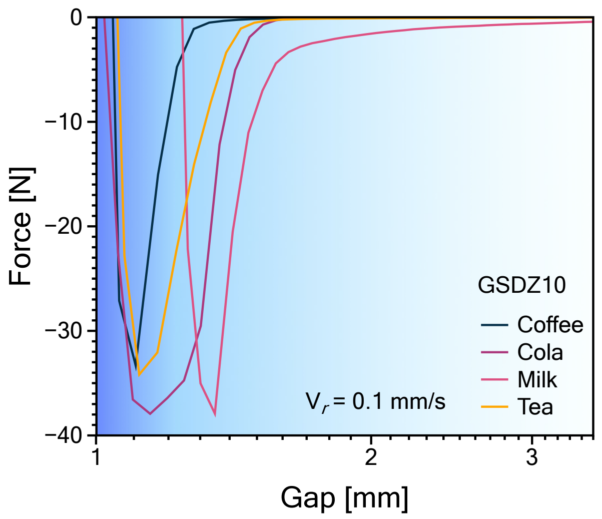

Figure S8. Force-retraction displacement curves at retraction speeds of 0.1 mm/s obtained for GSDZ10 samples after being dipped into different types of drinks. Note that in contrast to the data in Figure 5 where the absolute value of the force is represented, here the raw force values as recorded by the rheometer are presented.

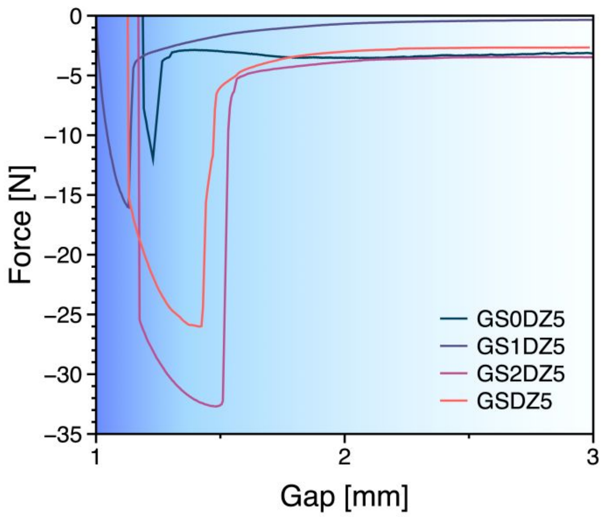

Figure S9. Force-retraction displacement curves at retraction speeds of 0.1 mm/s obtained for GS0DZ5, GS1DZ5, GS2DZ5 and GSDZ5. Note that in contrast to the data in Figure 5 where the absolute value of the force is represented, here the raw force values as recorded

by the rheometer are presented.

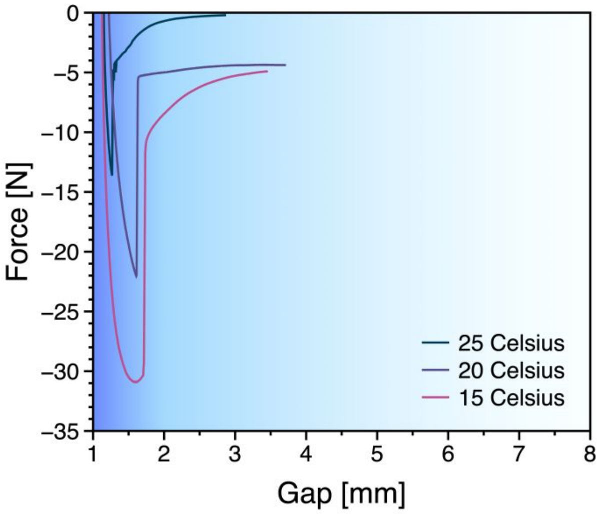

Figure S10. Force-retraction displacement curves at different temperatures with retraction speeds of 0.1 mm/s obtained for GSDZ5. Note that in contrast to the data in Figure 5 where the absolute value of the force is represented, here the raw force values as recorded by the rheometer are presented.

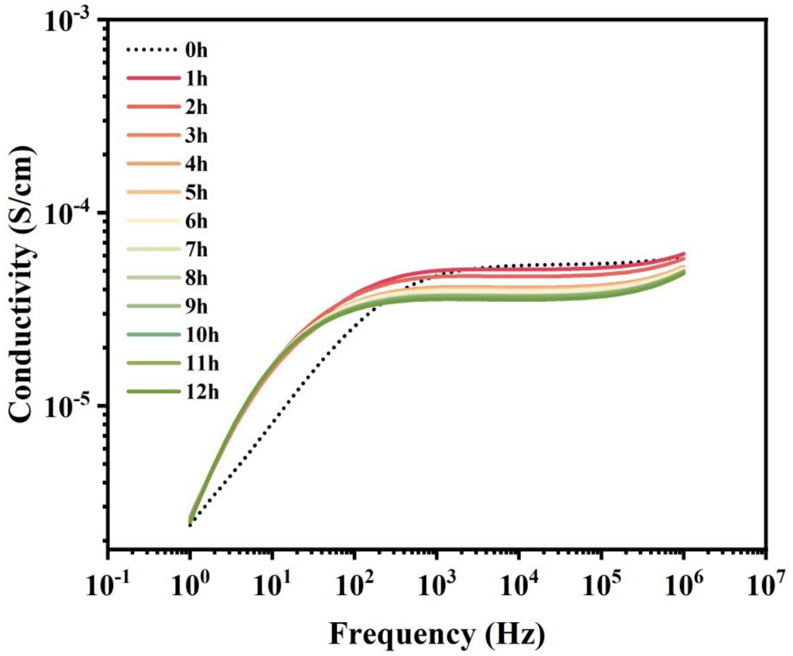

Figure S11. Dielectric spectra of GSDZ5 sample within 12 h. Before the measurement, the sample was immersed in water for a few seconds to make the whole surface wet.

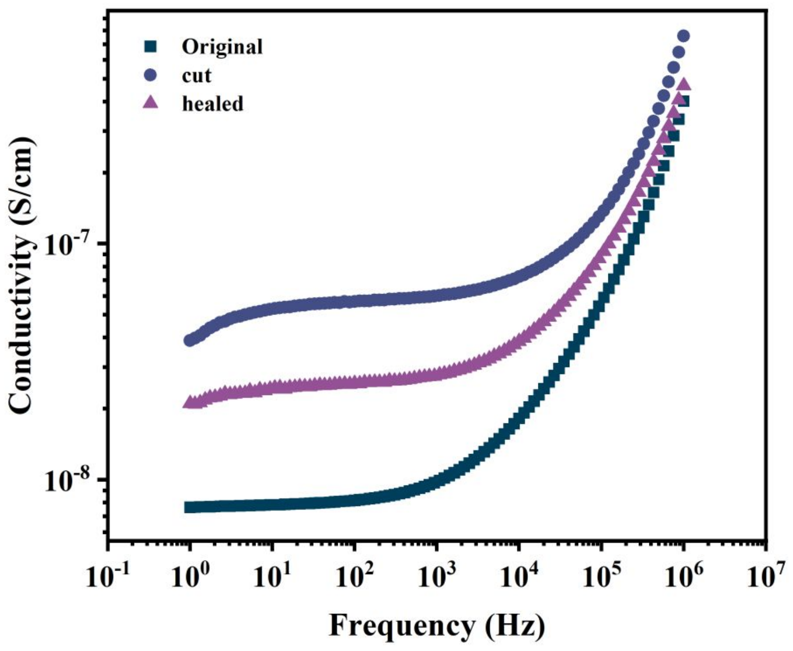

Figure S12. Dielectric spectra of GS1DZ5 sample before and after 12h self-healing.

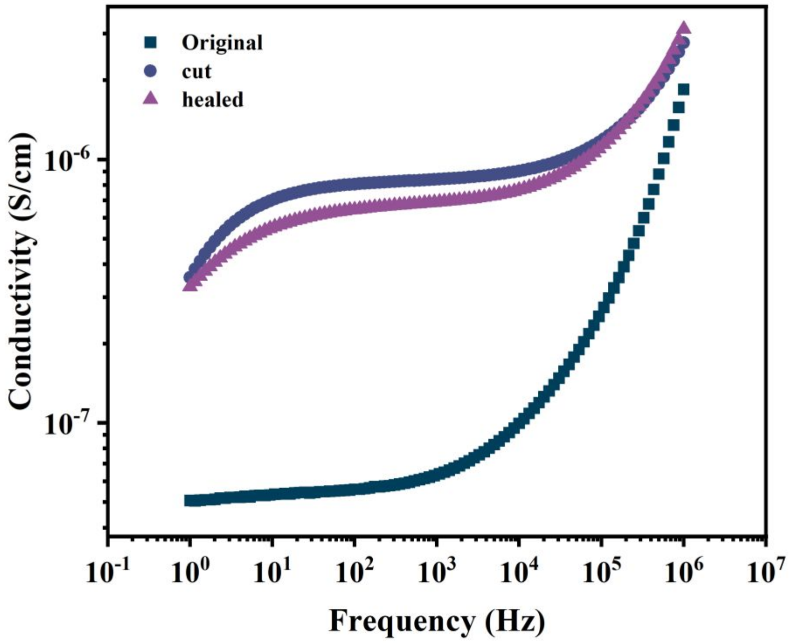

Figure S13. Dielectric spectra of GS2DZ5 sample before and after 12h self-healing.

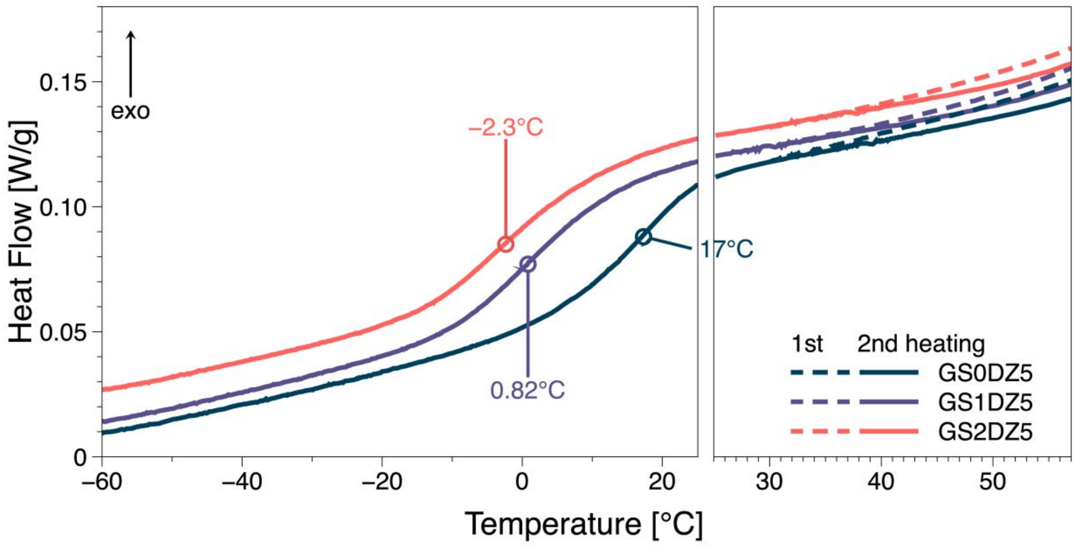

Figure S14. DSC curves of GS0DZ5, GS1DZ5 and GS2DZ5 hydrogels

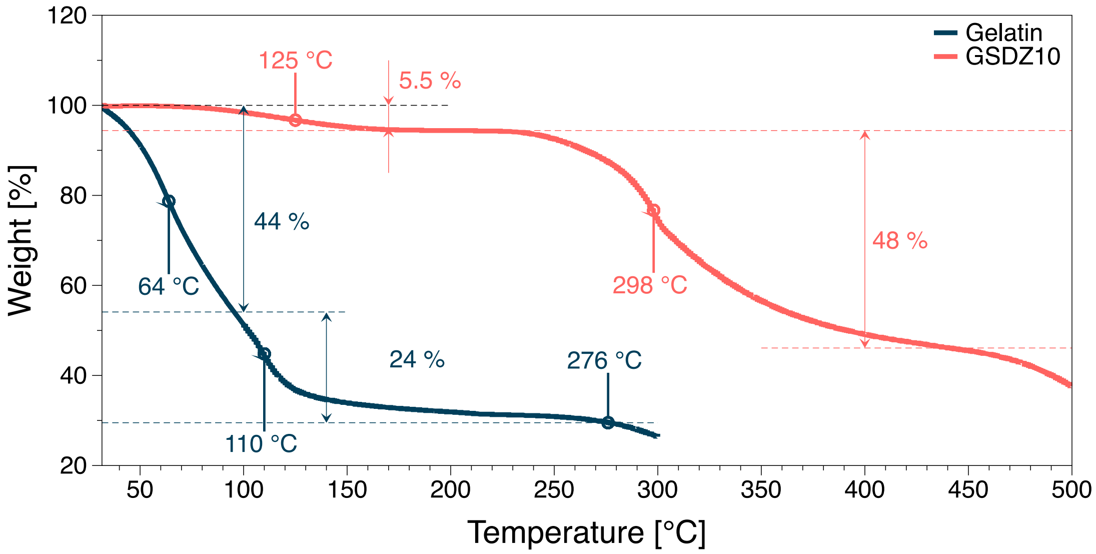

Figure S15. TGA curves of gelatin and GSDZ10 hydrogels.

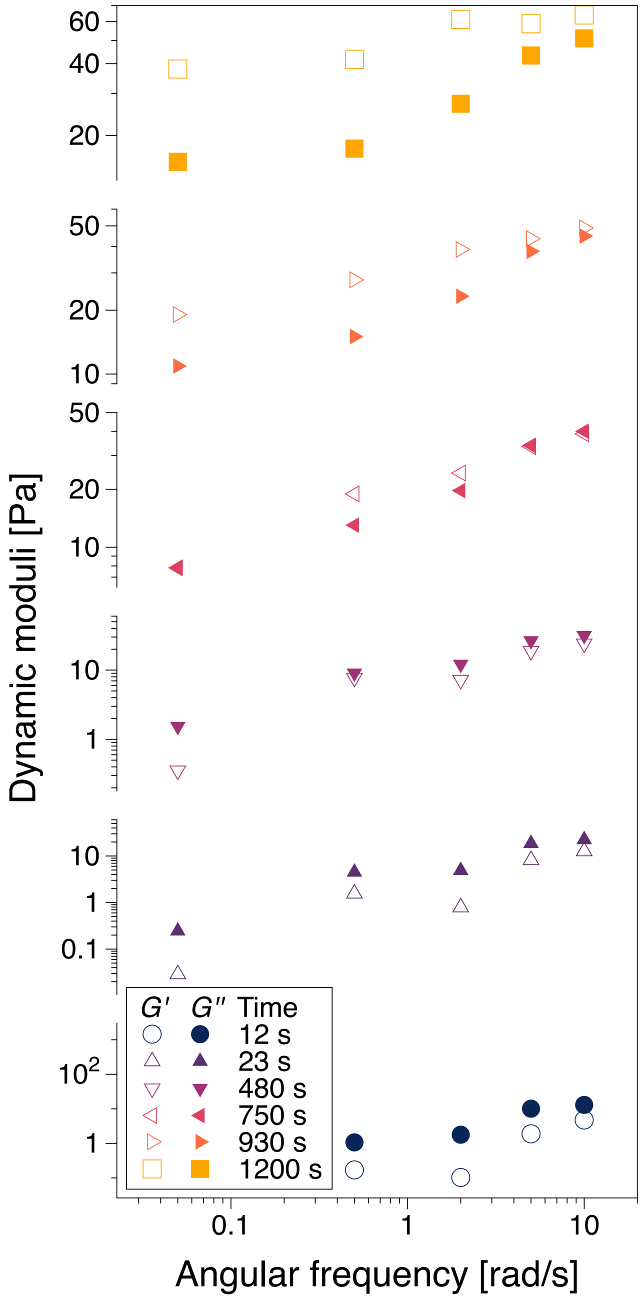

Figure S16. Dynamic moduli at several angular frequencies based on isochronous data.

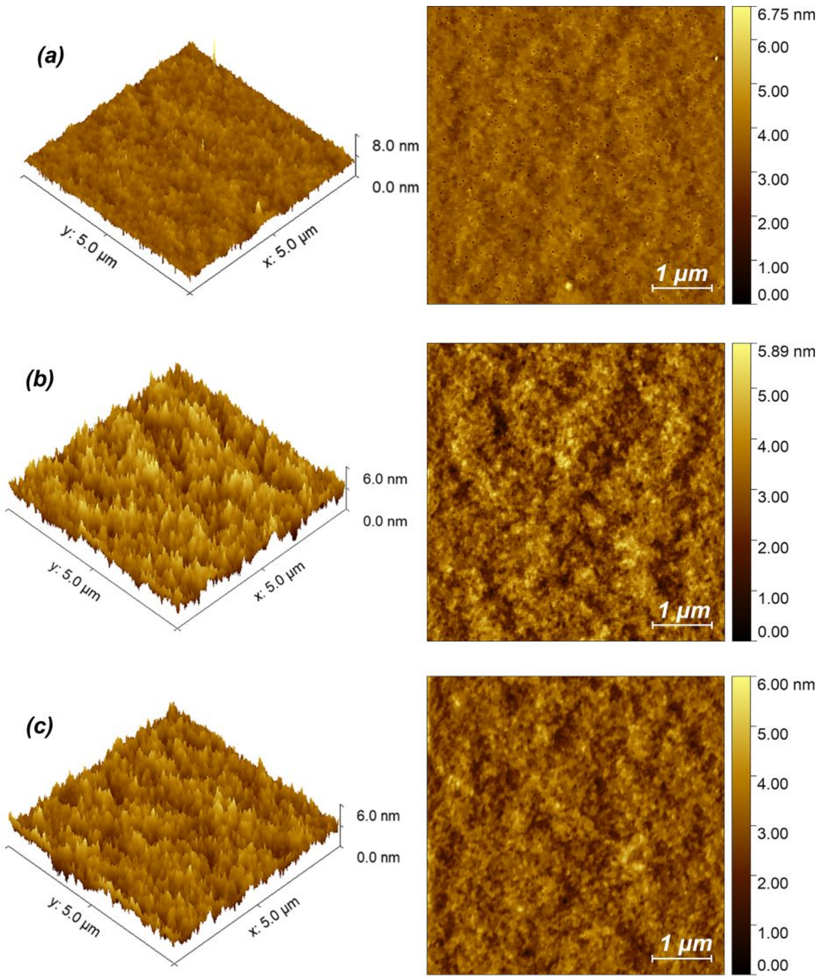
(a)
8.0 nm
0.0 nm
y: 5.0 µm
x: 5.0 µm
1 µm
6.75 nm
6.00
5.00
4.00
3.00
2.00
1.00
0.00
(b)
6.0 nm
0.0 nm
y: 5.0 µm
x: 5.0 µm
1 µm
5.89 nm
5.00
4.00
3.00
2.00
1.00
0.00
(c)
6.0 nm
0.0 nm
y: 5.0 µm
x: 5.0 µm
1 µm
6.00 nm
5.00
4.00
3.00
2.00
1.00
0.00

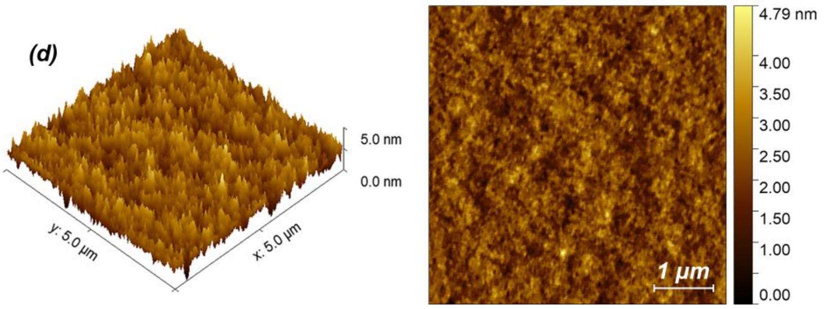

Figure S17. AFM images of bare glass (a), GSDZ5 (b), GSDZ10 (c) and GSDZ20 (d).
